# Interfacial Fe_5_C_2_-Cu catalysts toward low-pressure syngas conversion to long-chain alcohols

**DOI:** 10.1038/s41467-019-13691-4

**Published:** 2020-01-03

**Authors:** Yinwen Li, Wa Gao, Mi Peng, Junbo Zhang, Jialve Sun, Yao Xu, Song Hong, Xi Liu, Xingwu Liu, Min Wei, Bingsen Zhang, Ding Ma

**Affiliations:** 1 State Key Laboratory of Chemical Resource Engineering, Beijing Advanced Innovation Center for Soft Matter Science and Engineering, Beijing University of Chemical Technology, Beijing, 100029 P. R. China; 2 Beijing National Laboratory for Molecular Sciences, College of Chemistry and Molecular Engineering and College of Engineering, and BIC-ESAT, Peking University, Beijing, 100871 China; 3 State Key Laboratory of Coal Conversion, Institute of Coal Chemistry Chinese Academy of Sciences Taiyuan, Shanxi, 030001 P. R. China; 4 Synfuels China Beijing, 100195 Beijing, P. R. China; 5 Shenyang National Laboratory, Institute of Metal Research, Chinese Academy of Sciences, Shenyang, Beijing 100871 P. R. China

**Keywords:** Catalytic mechanisms, Heterogeneous catalysis, Chemical engineering

## Abstract

Long-chain alcohols synthesis (LAS, C_5+_OH) from syngas provides a promising route for the conversion of coal/biomass/natural gas into high-value chemicals. Cu-Fe binary catalysts, with the merits of cost effectiveness and high CO conversion, have attracted considerable attention. Here we report a nano-construct of a Fe_5_C_2_-Cu interfacial catalyst derived from Cu_4_Fe_1_Mg_4_-layered double hydroxide (Cu_4_Fe_1_Mg_4_-LDH) precursor, *i.e*., Fe_5_C_2_ clusters (~2 nm) are immobilized onto the surface of Cu nanoparticles (~25 nm). The interfacial catalyst exhibits a CO conversion of 53.2%, a selectivity of 14.8 mol% and a space time yield of 0.101 g g_cat_^−1^ h^−1^ for long-chain alcohols, with a surprisingly benign reaction pressure of 1 MPa. This catalytic performance, to the best of our knowledge, is comparable to the optimal level of Cu-Fe catalysts operated at much higher pressure (normally above 3 MPa).

## Abstract (web summary)

Long-chain alcohols synthesis from syngas conversion is a promising route for the production of high-value chemicals. Here the authors show that a heterogeneous Fe_5_C_2_/Cu catalyst derived from layered double hydroxides precursor exhibits excellent performance with a space time yield of 0.101 g g_cat_^−1^ h^−1^ at a low pressure of 1 MPa.

## Introduction

Long-chain alcohols, containing more than five carbons, are key alternative fuels and feedstock to manufacture plasticizers, detergents, and lubricants^1–4^. Synthesis of long-chain alcohols (LAS) from syngas by a tandem strategy provides a facile, economical, and environment-friendly approach^5–7^. Among various binary catalyst systems (Cu–Fe^1,3,8^, Cu–Co^9,10^, Co–Mo^11,12^), Cu–Fe binary candidates have attracted considerable attention in the production of long-chain alcohols. Previous studies on Cu–Fe binary catalysts show that a yield of long-chain alcohol as high as 0.014–0.144 g g_cat_^−1^ h^−1^ could be reached, albeit a relatively harsh reaction condition (3–8 MPa) is normally required. However, there is still room for the optimization of long-chain alcohols selectivity; more importantly, it is pivotal to develop catalyst system that works under mild reaction conditions^9–22^. Especially, the low pressure in practical operation gives a significant reduction in pressure drop, energy, and facility costs, which takes merits of both environmental and economic benefits^23,24^.

To achieve this goal, a precise control over the type, dimension, and nature of Cu/Fe interface plays a key role in the production of LAS. Maintaining a high degree of Cu–Fe interface is decisive for shifting the products from hydrocarbons to long-chain alcohols. We have demonstrated previously that Fe_5_C_2_ is very active for CO dissociation and C–C bond propagation, and thus is an excellent catalyst for the FTS reaction^25–27^. Lu et al. recently synthesized a 3DOM FeCu catalyst with atomic steps on the Cu surface involving planar defects and lattice strain, which showed excellent performance toward higher alcohols synthesis^3,16^. If we can prepare a highly dispersed iron carbide species over Cu, where CO is activated but not dissociated, it is possible to largely enhance the density of interfacial sites. This may render an optimized rate of C–C bond propagation on iron carbide sites and CO insertion on Cu/Fe_5_C_2_ interfacial sites, and thus a high selective toward long-chain alcohols would be achieved. Layered double hydroxides (LDHs), with unique structure that metal cations are distributed in the hydroxide layers at an atomic level, have attracted extensive attention as catalyst precursors for higher alcohols synthesis^10,15,18,28,29^.

In this report, we used Cu–Fe–LDHs as a precursor. After a structural topological transformation followed by an activation treatment^30–34^, Fe_5_C_2_ cluster supported on Cu particle catalyst is obtained. Fe_5_C_2_ clusters (~2 nm) are highly dispersed over the surface of Cu nanoparticles (~25 nm) which creates rich interfacial sites; and the Cu_4_Fe_1_ catalyst with optimized Fe_5_C_2_–Cu interface exhibits a CO conversion of 53.2% and space time yield of 0.101 g g_cat_^−1^ h^−1^ for long-chain alcohols, at a low operation pressure (1 MPa). This is even comparable with the optimal space time yield level of other Cu–Fe binary catalysts at pressure of 3–8 MPa.

## Results and discussion

### Catalysts synthesis and characterizations

**Fig. 1:**
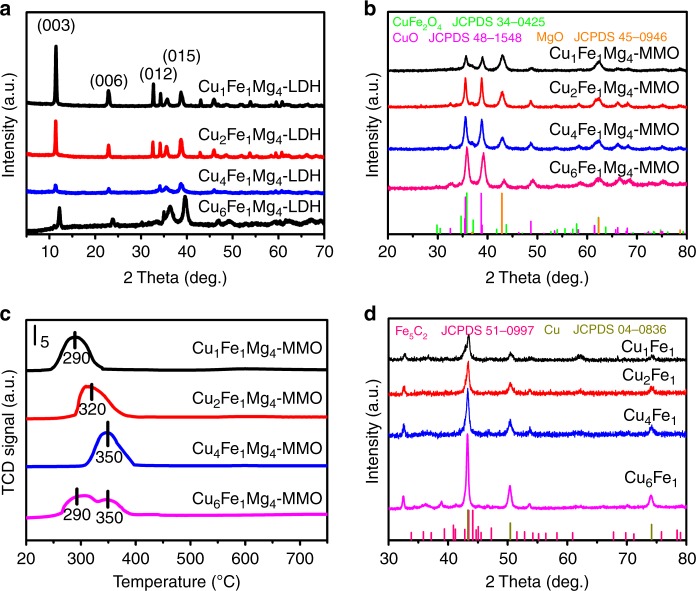
Catalysts characterizations. **a** XRD patterns of Cu_*x*_Fe_*y*_Mg_4_–LDH precursors with three molar ratios of Cu/Fe (1:1, 2:1, 4:1, and 6:1, respectively). **b** XRD patterns of Cu_*x*_Fe_*y*_Mg_4_–MMO calcined samples obtained from calcination of LDHs precursors. **c** H_2_-TPR profiles of Cu_*x*_Fe_*y*_Mg_4_–MMO samples. **d** XRD patterns of Cu_*x*_Fe_*y*_ obtained from activation of MMO samples in syngas (25% CO+25% H_2_+50% CO_2_) under optimum conditions (300 °C (2 h)+350 °C (1 h); rate: 2 °C min^−1^) and passivation.

The Cu_*x*_Fe_*y*_Mg_4_–LDH precursors with different Cu/Fe molar ratios (1/1, 2/1, 4/1, and 6/1, respectively) were prepared by a nucleation and aging separation method developed previously^15^. The XRD patterns (Fig. 1a) show characteristic diffractions corresponding to an LDH phase (JCPDS 14-0281); SEM images display a typical plate-like hexagonal morphology (Supplementary Fig. 1). Actually, Cu_6_Fe_1_Mg_4_–LDH could hardly give an LDH phase due to the strong Jahn–Teller effect of Cu^2+^. The LDHs precursors were transformed to mixed metal oxides (MMO) that inherit the plate-like morphology (Supplementary Fig. 2) after thermal treatment. A CuO phase is predominant for these three samples (Fig. 1b); while the diffractions at 35.8° and 37.1° confirm the formation of CuFe_2_O_4_ spinel in XRD patterns. From HRTEM images (Supplementary Fig. 3), some nanoparticles uniformly embedded in the platelet matrix are clearly observed. The CuFe_2_O_4_ nanoparticles are well-dispersed near CuO nanoparticles. According to the H_2_-TPR measurements (Fig. 1c), the main peak shifts gradually from 280 °C to 350 °C with the increase of Cu/Fe ratio, which is ascribed to the enhanced amount of CuFe_2_O_4_ spinel (a high reduction temperature) within CuO matrix (a low reduction temperature)^35–37^, as confirmed by XRD (Fig. 1b) and HRTEM (Supplementary Fig. 3). The Cu_4_Fe_1_Mg_4_–MMO shows the strongest interaction between CuO and CuFe_2_O_4_ species in accordance with HRTEM, possibly providing the most abundant and stable interfacial structure after activation. The precursors were reduced in syngas under optimized conditions to get the supported catalysts, which were denoted as Cu_*x*_Fe_*y*_ (*x*/*y* means the ratio between Cu and Fe). The Cu_*x*_Fe_*y*_ catalysts show similar XRD patterns (Fig. 1d) with metallic Cu. The average Cu particle sizes are 15.7, 18.4, 21.7, and 25.1 for Cu_1_Fe_1_, Cu_2_Fe_1_, Cu_4_Fe_1_, and Cu_6_Fe_1_ samples, respectively (Supplementary Table 1). Moreover, no obvious diffraction of Fe crystalline is resolved, which indicates that Fe species is highly dispersed in the catalyst.

### Catalytic results

**Fig. 2:**
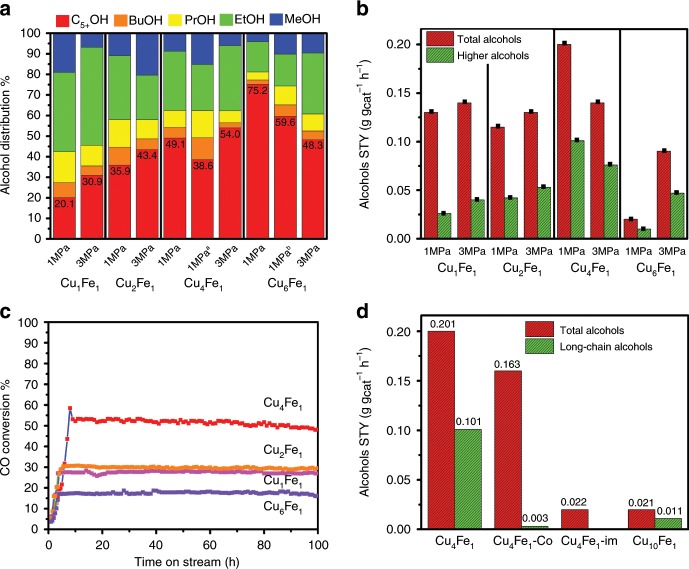
Catalytic performance. **a** Alcohol distribution normalized data to total alcohols selectivity (^a^ WHSV = 4800 mL g_cat_^−1^ h^−1^; ^b^ WHSV=1200 mL g_cat_^−1^ h^−1^; C_5+_OH: long-chain alcohols, BuOH: butanol, PrOH: propanol, EtOH: ethanol and MeOH: methanol) and **b** alcohols STY at different pressures over catalysts with four Cu/Fe ratios (1/1, 2/1, 4/1, and 6/1) (reaction conditions: 27% CO+55% H_2_+18% N_2_; 260 °C; WHSV of 2400 mL g_cat_^−1^ h^−1^). **c** Time-on-stream (TOS) evolution of CO conversion over the four catalysts within 100 h test at 1 MPa. **d** Alcohols STY of Cu_4_Fe_1_, Cu_4_Fe_1_-co, Cu_4_Fe_1_-im, and Cu_10_Fe_1_ at 1 MPa.

The catalysts were first evaluated under a relatively high pressure (3 MPa). Figure 2a shows the alcohol distribution over catalysts with various activation treatments (Supplementary Tables 2–6), from which both the activation atmosphere (syngas containing CO_2_) and the two-stage activation procedure play a vital role in tuning the synergistic effect between Cu and Fe_5_C_2_. The sample activated in syngas containing CO_2_ (Supplementary Table 4) could slow down the rate of reduction process and facilitate carburization of Fe to produce Fe_5_C_2_ species, which would maintain the tiny size of Fe_5_C_2_ to form abundant Cu–Fe_5_C_2_ interfacial sites. The sample reduced in syngas shows diffraction peaks of both Cu and Fe_5_C_2_ phase (Supplementary Fig. 4) and rather poor catalytic behavior for the production of LA, indicating less interfacial sites merged by poor dispersion of Fe_5_C_2_ is not beneficial for the production of LA. The selectivity toward long-chain alcohols enhances gradually along with the increase of Cu/Fe ratio, reaching maximum at 54.0% for Cu_4_Fe_1_ at 3 MPa (specific data in Supplementary Table 7). Cu_4_Fe_1_ gives out the optimal space time yield (STY) (Supplementary Table 8) toward total alcohols (0.141 g g_cat_^−1^ h^−1^) and long-chain alcohols (0.076 g g_cat_^−1^ h^−1^) at 3 MPa.

What attracts our attention most is the excellent long-chain alcohols production on Cu_4_Fe_1_ under 1 MPa (Fig. 2; Supplementary Fig. 5). The Cu_4_Fe_1_ has 38.6% at WHSV = 4800 mL g_cat_^−1^ h^−1^ with iso-conversion to other Cu_*x*_Fe_*y*_ samples, although Cu_4_Fe_1_ has higher value. The total alcohols yield (0.201 g g_cat_^−1^ h^−1^) of Cu_4_Fe_1_ is much higher than hydrocarbons yield (0.111 g g_cat_^−1^ h^−1^), demonstrating the predominant production of alcohols rather than hydrocarbons (Supplementary Table 8). This means the high LA yield is the interplay of activity and selectivity. In addition, long-chain alcohols (C_5+_ alcohols) give a higher yield than methanol (0.101 vs. 0.018 g g_cat_^−1^ h^−1^), which is among the highest level compared with previous work in Supplementary Table 9. Normally, high reaction pressure (3–8 MPa) is required for long-chain alcohols synthesis; in this work, however, a low-pressure (1 MPa) syngas conversion to long-chain alcohols is attained. Significantly, Cu_4_Fe_1_ catalyst at 1 MPa reaction condition shows a satisfactory stability: 5% decrease in activity is observed within a 100 h catalytic evaluation test (Fig. 2c) and a satisfactory reproducibility (Supplementary Table 10).

To understand the outstanding catalytic performance of Cu_4_Fe_1_, the impact of catalyst synthesis on its catalytic performance was investigated. Control samples with the same Cu/Fe ratio of 4/1 synthesized through conventional impregnation and co-precipitation methods were prepared (Supplementary Fig. 6: termed as Cu_4_Fe_1_-im and Cu_4_Fe_1_-co, respectively), from which Cu–Fe_5_C_2_ interfaces were rarely observed over those conventional catalysts. However, both catalysts produced scarcely any long-chain alcohols (Supplementary Table 11), indicating the unique interfacial structure derived from the LDHs synthesis route definitely changes the catalytic activity. Another two control samples of pure Cu (denoted as Cu_4_) or pure Fe (denoted as Fe_1_) were prepared based on a similar LDHs approach. The Cu_4_ sample mainly shows methanol synthesis performance and the Fe_1_ gives conventional FTS performance (Supplementary Table 11), in accordance with previous work^38–40^. When separate Cu_4_ and Fe_1_ catalysts were combined with various modes, long-chain alcohols could not be synthesized. This indicates that the Cu–Fe_5_C_2_ synergistic effect is responsible for the production of long-chain alcohols. Therefore, the synergy of interfacial sites between Fe_5_C_2_ and Cu is must for the long-chain alcohols (higher alcohols) synthesis from syngas. We performed further studies on samples with higher Cu/Fe molar ratios as 10:1 (termed as Cu_10_Fe_1_, Supplementary Fig. 7). Although both Cu_6_Fe_1_ and Cu_10_Fe_1_ samples have good selectivity of long-chain alcohols (Supplementary Table 12), the STY was low (Fig. 2b, d), showing that a suitable Cu/Fe ratio is important for the best catalytic performance. This suggests that catalyst with rich Cu–Fe_5_C_2_ interfaces derived from the LDHs precursor method could result in an excellent activity and selectivity toward long-chain alcohols even at a low reaction pressure.

### Fine structure characterizations

**Fig. 3:**
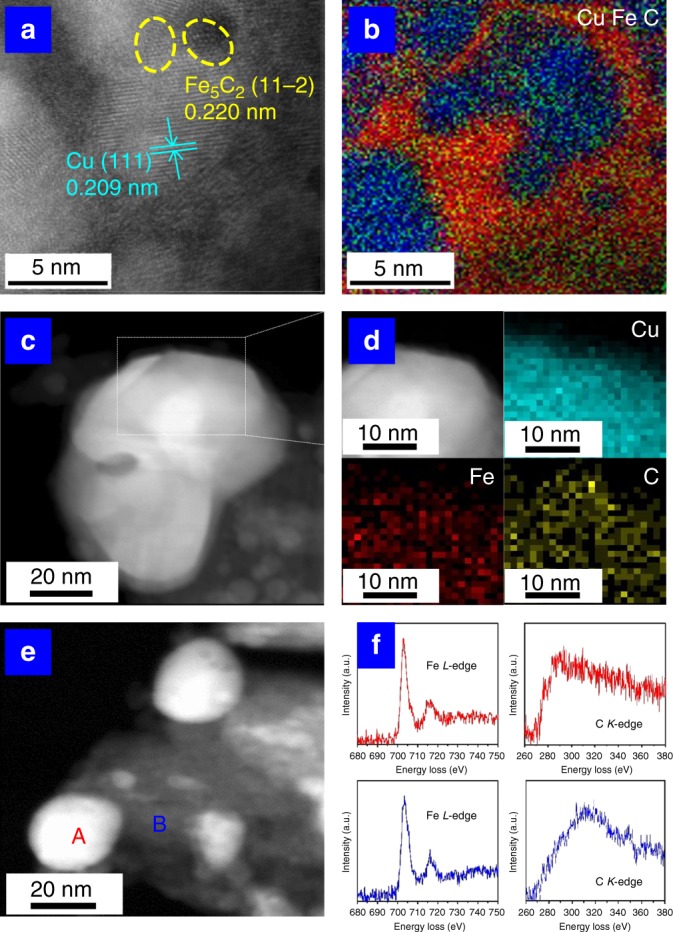
Structural studies on Cu_4_Fe_1_ sample. **a** HRTEM image; **b** EELS mapping of Cu (blue), C (red), and Fe (green) of (**a**). quasi-in-situ STEM: **c** HAADF-STEM image; **d** EDS mapping of the selected region (white box in **c**) showing elemental distribution of Cu, C, and Fe; **e** HAADF-STEM image and **f** EELS curves of Fe L-edge and C K-edge selected from (**e**) (Spot A: red, Spot B: blue).

To further understand the structural property of Cu_*x*_Fe_*y*_ catalysts, the three samples (Cu_1_Fe_1_, Cu_2_Fe_1_, and Cu_4_Fe_1_) were characterized by aberration-corrected scanning transmission electron microscopy (ac-STEM). For Cu_1_Fe_1_ and Cu_2_Fe_1_ samples (Supplementary Figs. 8, 9), the lattice fringe of Cu (111) with 0.209 nm is clearly resolved; EDS mapping images of Cu, Fe, and C demonstrate the existence of Fe_5_C_2_ nanoclusters on the surface of Cu nanoparticles. In the case of Cu_4_Fe_1_ sample, Fig. 3a shows the lattice fringe of Cu (111) and Fe_5_C_2_ (11-2); corresponding EELS mapping (Fig. 3b) illustrates the modification of Cu by Fe_5_C_2_ nanoclusters within a single Cu nanoparticle, which is also confirmed in another selected region in Supplementary Fig. 10. Then, quasi-in-situ STEM measurements on the used catalyst after 1 MPa syngas conversion reaction were performed (Fig. 3). The EDX-mapping results (Fig. 3c, d) also verified the homogeneous distribution of iron species on Cu. The phase of the iron species could be identified to be Fe_5_C_2_, as the catalyst shows sextets characteristics in Mössbauer spectrum (Supplementary Fig. 11)^16,41^. To further confirm the existence of iron carbide species on Cu particles, the electron energy-loss spectra (EELS) of C K-edge at the surface of Cu particle (Spot A) and support (Spot B) were studied (Fig. 3e, f). For C K-edge spectra, Spot A displays a lower energy peak relative to Spot B, accompanied by a small peak at ~280 eV^42^, which again verifies the existence of carbide species on the surface of Cu particle.

**Fig. 4:**
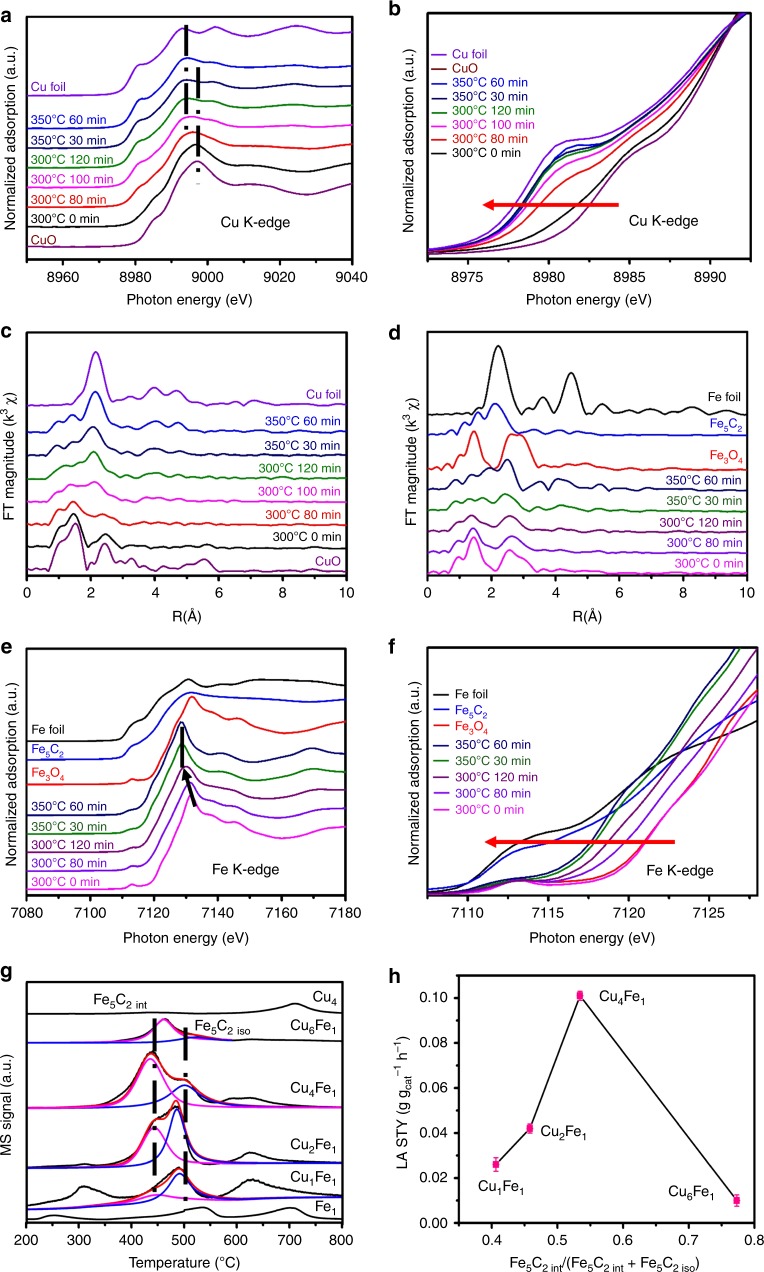
In situ XAS measurements of Cu_4_Fe_1_ catalyst and CO-TPD measurements of Fe_1_, Cu_4_ and Cu_*x*_Fe_*y*_ samples. **a**, **e** In situ XANES spectra at Cu K-edge and Fe K-edge of Cu_4_Fe_1_Mg_4_-MMO in syngas treatment to obtain Cu_4_Fe_1_ catalyst (5% CO + 5% H_2_ + 10% CO_2_ + 80% He stream; 20 mL min^−1^) at different temperatures and time points, respectively. **b**, **f** Enlarged view of absorption edge selected from (**a**, **e**). **c**, **d** Fourier transform magnitude of k^3^-weighted in situ Cu K-edge and Fe K-edge EXAFS spectra, respectively. **g** CO-TPD profiles of Fe_1_, Cu_4_, and Cu_*x*_Fe_*y*_ samples. **h** Long-chain alcohols space time yield as a function of Fe_5_C_2int_/(Fe_5_C_2int_ + Fe_5_C_2iso_). Fe_5_C_2int_: interfacial Fe_5_C_2_; Fe_5_C_2iso_: isolated Fe_5_C_2_. The error bars are between 0.002 and 0.003.

In addition, in situ X-ray absorption near-edge structure (XANES) and extended X-ray absorption fine structure (EXAFS) experiments for Cu_4_Fe_1_Mg_4_–MMO at Cu K-edge and Fe K-edge (Fig. 4a, f) were performed to reveal the structural change during the activation treatment process to obtain Cu_4_Fe_1_ catalyst (in 20 mL min^−1^ 5% CO+5% H_2_+10% CO_2_+80% He stream). The normalized Cu K-edge XANES spectra show a gradual decrease in the energy of absorption edge relative to CuO reference, accompanied with the appearance of edge features of metallic Cu, indicating a progressive reduction of CuO phase to metallic Cu. Corresponding fitting results (Supplementary Fig. 12) show that Cu species in Cu_4_Fe_1_ is mainly metallic Cu with rather small amount of CuO_*x*_. Based on the results of previous reports^43,44^ and observations in this work, Cu^0^ is regarded as active site for CO adsorption/insertion. In the Cu K-edge EXAFS spectra, initially, the coordination environment of Cu_4_Fe_1_Mg_4_–MMO is consistent with CuO, in which the peaks at 1.5 Å and 2.5 Å are assigned to the first Cu–O coordination and Cu–Cu coordination, respectively^45,46^. During the activation process, the peak at 2.2 Å ascribed to Cu–Cu coordination shell gradually emerges and becomes predominant, further confirming the reduction to metallic Cu. In the case of Fe species, the normalized Fe K-edge XANES spectra show the reduction of CuFe_2_O_4_ spinel to iron carbide phase along with the absorption edge moving to low energy. As shown in Fe K-edge EXAFS spectra, Fe–Fe coordination shell at 2.0 Å is observed at 350 °C, confirming the formation of iron carbide^3,47,48^. Based on above results, it is concluded that the new interfacial structure is well established from LDHs precursor through refined activation process.

### Structure-performance relationship

In addition, CO-TPD (Fig. 4g, h) was performed to quantitatively study the amount of Fe_5_C_2_ interfacial sites. As CO adsorption on Cu is rather weak and only be resolved through low-temperature TPD^49,50^, the observed desorption peaks are ascribed to iron species. In addition to the CO desorption from iron oxide at relatively low temperature (around 300 °C, for Cu_1_Fe_1_) and reverse Boudouard reaction (above 600 °C), two desorption peaks at 440 °C and 490 °C (for Cu_1_Fe_1_, Cu_2_Fe_1_, Cu_4_Fe_1_, and Cu_6_Fe_1_) could be attributed to CO desorption from iron carbide^51,52^. With Fe_1_ as a reference, these two peaks (440 °C and 490 °C) are attributed to interfacial Fe_5_C_2_ (Fe_5_C_2int_) and isolated Fe_5_C_2_ (Fe_5_C_2iso_) species, respectively. Clearly, Cu_4_Fe_1_ gives the largest integral peak area, indicating the most abundant total Fe_5_C_2_ sites than other samples. We fitted and deconvoluted the two peaks to roughly estimate the ratio of Fe_5_C_2int_/(Fe_5_C_2int_+Fe_5_C_2iso_) for these four Cu_*x*_Fe_*y*_ catalysts, and the results showed a volcanic correlation between the LA yield and the relative concentration of interfacial Fe_5_C_2_ (Fig. 4h). Cu_4_Fe_1_ catalyst with a moderate Fe_5_C_2int_ ratio possesses the highest concentration of Fe_5_C_2_–Cu interface sites (Fe_5_C_2int_), accounting for the largest LA yield (0.101 g g_cat_^−1^ h^−1^). This demonstrates that the Fe_5_C_2_–Cu interfacial sites act as active center toward LA production.

It is well-known that CO does not dissociate over Cu; while Fe_5_C_2_ (or iron carbide in general) is beneficial for CO dissociation and subsequent C–C bond propagation^25–27,47,53^. The unique interfacial structure of ultrasmall Fe_5_C_2_ clusters over Cu particles has conferred the Cu_4_Fe_1_ catalyst a suitable construct for the production of long-chain alcohols production at syngas pressure as low as 1 MPa. De Jong et al. reported the size effect in supported iron carbides (2–7 nm) for Fischer–Tropsch reaction, in which a smaller particle size improved the coverage of CH_*x*_ species (the monomer of carbon chain growth)^54,55^. In this work, long-chain alcohols synthesis at 1 MPa was achieved, which was described to the unique structure of Cu_4_Fe_1_ catalyst. The Fe_5_C_2_ nanoclusters, with an ultrasmall size (normally below 2 nm) on the surface of Cu nanoparticles, provide active sites for CO activation/dissociation and the resulting C–C bond propagation, which maintains the high activity of Cu_4_Fe_1_. As the reaction pressure decreases from 3 MPa to 1 MPa, hydrogen activation on catalyst surface is weakened (Supplementary Fig. 13), which reduces the rate of hydrogenation and hydrocarbons chain termination and thus enhances the growth of carbon chain. In contrast, the CO activation is promoted since CO molecule is prone to adsorb on a Cu-rich surface^56,57^. This facilitates the kinetic rate coordination between CO insertion and C–C coupling, and therefore significantly elevates the selectivity toward total alcohols and long-chain alcohols^58–60^. This is also verified by the *α*-ASF chain-lengthening probabilities analysis: the *α*-value for alcohols (0.72) at 1 MPa exceeds that for hydrocarbons (0.70), indicating the rate of CO insertion is larger than that of hydrocarbons chain termination (Supplementary Fig. 4). Therefore, a precise control over double-active-site in Cu_4_Fe_1_ catalyst accounts for the high yield toward long-chain alcohols at 1 MPa.

In summary, Cu_4_Fe_1_ catalyst was synthesized from LDH precursor which exhibits a CO conversion of 53.2% and long-chain alcohols space time yield of 0.101 g g_cat_^−1^ h^−1^, at a surprisingly benign pressure of 1 MPa. Combination of study verifies that highly dispersed Fe_5_C_2_ nanoclusters are confined over the surface of Cu nanoparticles, forming abundant Fe_5_C_2_–Cu interfacial sites; and the synergistic interaction between Fe_5_C_2_ clusters and Cu nanoparticle is responsible for the unique catalytic performance toward long-chain alcohol production at low pressure. This work not only discovers a catalyst with unique structure for LAS but also sheds light on the construct of binary metal catalyst.

## Methods

### Catalysts synthesis

The Cu_*x*_Fe_*y*_Mg_4_-LDH precursors with four Cu:Fe:Mg molar ratios of 1:1:4, 2:1:4, 4:1:4, and 6:1:4 (denoted as Cu_1_Fe_1_Mg_4_-LDH, Cu_2_Fe_1_Mg_4_-LDH, Cu_4_Fe_1_Mg_4_-LDH, and Cu_6_Fe_1_Mg_4_-LDH) were prepared by using a method of nucleation and aging separation developed in our laboratory^12^. Solution A was a mixture of Cu(NO_3_)_2_·3H_2_O, Fe(NO_3_)_3_·9H_2_O, and Mg(NO_3_)_2_·6H_2_O with various Cu:Fe:Mg molar ratios dissolved in 100 mL of deionized water ([Cu^2+^] + [Mg^2+^] + [Fe^3+^] = 1.0 M). Solution B was obtained by dissolving NaOH and Na_2_CO_3_ in the same volume of deionized water with [NaOH] = 1.6 M and [CO_3_^2−^] = 2[Fe^3+^]. Solution A and B were simultaneously added to a colloid mill rotating at 4000 rpm and mixed for 2 min. The resulting slurry was removed from the colloid mill and aged at 120 °C for 24 h. The final precipitate was filtered, washed thoroughly with deionized water, and dried at 60 °C for 24 h.

General activation process: the Cu_*x*_Fe_*y*_Mg_4_-LDH precursors were calcined in air at 500 °C for 4 h at a heating rate of 2 °C min^−1^ to obtain mixed metal oxides (MMOs) (denoted as Cu_1_Fe_1_Mg_4_–MMO, Cu_2_Fe_1_Mg_4_–MMO, Cu_4_Fe_1_Mg_4_–MMO, and Cu_6_Fe_1_Mg_4_–MMO); subsequently, these MMOs materials were reduced in syngas atmosphere consisting of 25% CO+25% H_2_+50% CO_2_ with a two-step-process—300 °C for 2 h and 350 °C for another 1 h at a heating rate of 2 °C min^−1^ (denoted as Cu_1_Fe_1_, Cu_2_Fe_1_, Cu_4_Fe_1_, and Cu_6_Fe_1_, respectively).

In addition, based on Cu_4_Fe_1_Mg_4_-LDH precursor, the calcination temperature, activation procedure, and activation atmosphere were modulated to investigate their effects on catalytic performance, and the results were shown in Supplementary Tables 2–4. Cu_4_, Fe_1_, and Cu_10_Fe_1_ were prepared following above synthesis and activation process, which were derived from Cu_4_Al_1_Mg_4_–LDH, Fe_1_Mg_8_–LDH, and Cu_10_Fe_1_Mg_4_–LDH changing the ratio to Cu:Al:Mg = 4:1:4, Fe:Mg = 1:8, and Cu:Fe:Mg = 10:1:4, respectively (calcined samples: Cu_4_Al_1_Mg_4_–MMO, Fe_1_Mg_8_–MMO, and Cu_10_Fe_1_Mg_4_–MMO).

Cu_4_Fe_1_-co as a reference sample was prepared by co-precipitated method^10^. A mixture of copper and iron nitrate (Cu/Fe molar ratio of 4/1) was precipitated with an aqueous solution of Na_2_CO_3_. After aging for 4 h, the precipitate was washed thoroughly with distilled water until pH = 7–8, and was dried at 60 °C for 12 h. Then, the sample was calcined and reduced via the same procedure described above. Cu_4_Fe_1_-im as a reference sample was prepared by incipient-wetness impregnation method^9^. In all, 8.0 g of CuO powdered sample was added into an aqueous solution containing 0.025 M iron nitrate. After impregnation for 4 h, the catalyst precursor was dried in air at 60 °C for 12 h followed by calcination and reduction in general activation process.

### Catalytic evaluation

The catalytic evaluation was carried out in a 10 -mm fixed-bed stainless-steel reactor. In total, 1.0 g of MMO precursor was loaded on the catalyst bed, and the remaining volume of the reactor tube was filled with quartz beads of 20–40 mesh. Before reaction, the catalyst was activated in situ as mentioned above with a flow rate of 40 mL min^−1^. After the reactor was cooled to room temperature, syngas with a flow rate of 40 mL min^−1^ (27% CO+55% H_2_+18% N_2_) was introduced to purge the reaction line and reach the required pressure with nitrogen as an internal standard gas. The reaction was conducted at 260 °C. The outlet gas components (CO, H_2_, CH_4_, CO_2_, and N_2_) after passing through a hot trap (180 °C) and a cold trap (5 °C) were determined using an online GC-2014C Shimadzu gas chromatograph with TCD detector (TDX-1 column) and FID detector (Al_2_O_3_ packed column) using He as carrier gas. The liquid products were collected from the hot trap and cold trap, followed by determination offline with FID detector (RTX-5). 1,4-dioxane was used as internal standard for the aqueous products; and ethyl cyclohexane was used as an internal standard after extraction by cyclohexane.

1 $$\begin{array}{l}{\mathrm{CO}}\,{\mathrm{conversion}}\,{\mathrm{was}}\,{\mathrm{defined}}\,{\mathrm{as}}\!:\!{\mathrm{CO}}\,{\mathrm{conversion}}\left( \% \right)\\ = \frac{{{\it{F}}_{{\it{{\mathrm{CO}},\,{\mathrm{in}}}}} - {\it{F}}_{{\it{{\mathrm{CO}},\,{\mathrm{out}}}}}}}{{{\it{F}}_{{\it{{\mathrm{CO}},\,{\mathrm{in}}}}}}} \times 100\end{array}.$$

2 $${\mathrm{Product}}\,{\mathrm{selectivity}}\,{\mathrm{was}}\,{\mathrm{defined}}\,{\mathrm{as}}\!:\!{\mathrm{selectivity}}\left( {{\mathrm{mol}}\% } \right) = \frac{{{\it{F}}_{{\it{Ci}}}{\it{ \times i}}}}{{\sum {\it{F}}_{{\it{Ci}}}{\it{ \times i}}}} \times 100.$$

Where *F* is the moles of CO and product *Ci* (CO_2_, hydrocarbon, or alcohols) containing *i* carbon atoms. The mass balance and carbon balance have been calculated at each product and kept between 85 and 90%.

The ASF chain growth probability *α* is calculated according to the equation: ln (*W*_*n*_/*n*) = *n*ln*α* + ln(1 − *α*)^2^/*α*, where *n* is the number of carbon atoms in products; *W*_*n*_ is the weight fraction of products containing *n* carbon atoms; and 1 − *α* is the probability of chain termination.

### Catalyst characterization

Powder XRD measurements were performed on a Rigaku XRD-6000 diffractometer, using Cu Kα radiation (*λ* = 0.15418 nm) at 40 kv and 30 mA, with a scanning rate of 5° min^−1^ and a 2*θ* angle ranging from 3° to 90°. The phases of components were identified based on JCPDS standard cards. Scanning electron microscope (SEM; Zeiss SUPRA 55) with an accelerating voltage of 20 kV was performed. Aberration-corrected scanning transmission electron microscopy (ac-STEM), electron energy-loss spectroscopy (EELS), and element energy-dispersive spectroscopy (EDS) mapping measurements were carried out on a JEOL JEM-ARM200F instrument. The quasi-in-situ scanning transmission electron microscopy (STEM) and EDX-mapping measurements were performed on a FEI Tecnai G2 F20 microscope with an accelerating voltage of 120 kV. The sample was hold in glovebox in Ar atmosphere and transferred by a vacuum transfer TEM holder. The specific surface area determination and pore volume analysis were performed by Brunauer–Emmett–Teller (BET) and Barret–Joyner–Halenda (BJH) methods using a Quantachrome Autosorb-1C-VP Analyzer. Elemental analysis for Cu and Fe was performed using a Shimadzu ICPS-75000 inductively coupled plasma atomic emission spectrometer (ICP-AES). Hydrogen temperature-programmed reduction (H_2_-TPR) was measured on a Micromeritics ChemiSorb 2070 with a thermal conductivity detector (TCD). In a typical process, 100 mg of sample was sealed in a quartz tube reactor and pretreated in a Ar atmosphere at 150 °C for 2 h, followed by reduction in a stream of H_2_/Ar (1/9, v/v; a total flow rate of 40 mL min^−1^) at a heating rate of 10 °C min^−1^ up to 800 °C. Mössbauer spectrum experiments of the as-prepared catalysts were carried out at −268.8 °C. The spectrometer was calibrated using a standard *α*-Fe foil and was fitted with five sextets, which reflect Fe_5_C_2_ and Fe_3_O_4_ with different hyperfine parameters. The spectra components were identified according to their isomer shift, quadruple splitting, and magnetic hyperfine field. Magnetic hyperfine fields were calibrated with the 330 kOe field of *α*-Fe. In situ X-ray absorption fine structure spectroscopy (XAFS) at the Fe and Cu K-edge were acquired at 1W2 beamline of at Beijing Synchrotron Radiation Facility (BSRF) under transmission mode. The typical energy of the storage ring was 2.5 GeV with a maximum current of 250 mA. The Si (111) double-crystal monochromator was used. The powdered sample was first pressed into sheet and loaded into a reactor cell equipped with polyimide windows. The sample sheet was reduced in 5% CO+5% H_2_+10% CO_2_ + 80% He stream with 20 mL min^−1^, and underwent a heat treatment at 300 °C for 120 min then at 350 °C for 180 min (rate: 2 °C min^−1^). The XAFS spectra were collected 60 times h^−1^ in the whole process. All the collected spectra were processed and analyzed using Athena code within Ifeffit package. CO- or H_2_-temperature-programmed desorption (CO-TPD or H_2_-TPD) experiments were carried out in a fixed-bed reactor and detected by MS. 100 mg catalyst after reaction was exposed to CO or H_2_ for 120 min in room temperature. The catalyst was switched to He exposure until the baseline of the CO or H_2_ signal leveled off. Finally, the temperature was increased to 800 °C at 10 °C min^−1^. The mass signal of 28 or 2 was monitored by quadruple mass spectrometer. The apparent kinetic order of H_2_ was measured as follows: the catalytic test was performed at 260 °C with Cu_4_Fe_1_ catalyst (0.1 g) in a gas flow rate of 40 mL min^−1^ in order to keep the H_2_ conversion under 10%. For determining the order of H_2_, the partial pressure of H_2_ was controlled from 22.5 to 45%.

## Supplementary information

Supplementary Information

Peer Review

## Data Availability

The data underlying Figs. 1, 2, 4, Supplementary Figs. 4–6, 7a, c, 11, and 13 are provided as a Source Data file. The other data that support the findings of this study are available from the corresponding author upon request.

## Source data

Source Data
