# Supplementary material for: Interfacial Fe5C2-Cu catalysts toward low-pressure syngas conversion to long-chain alcohols
Source: Nat Commun. 2020 Jan 3;11:61. doi: 10.1038/s41467-019-13691-4 (PMC6941981; doi:10.1038/s41467-019-13691-4)
Supplement: Supplementary file 1 — Supplementary Information [file 41467_2019_13691_MOESM1_ESM.pdf]

Supplementary Information for

# Interfacial $Fe_5C_2$-Cu catalysts toward Low-Pressure Syngas Conversion to Long-Chain Alcohols

Li et al.

## Supplementary Figures and Tables

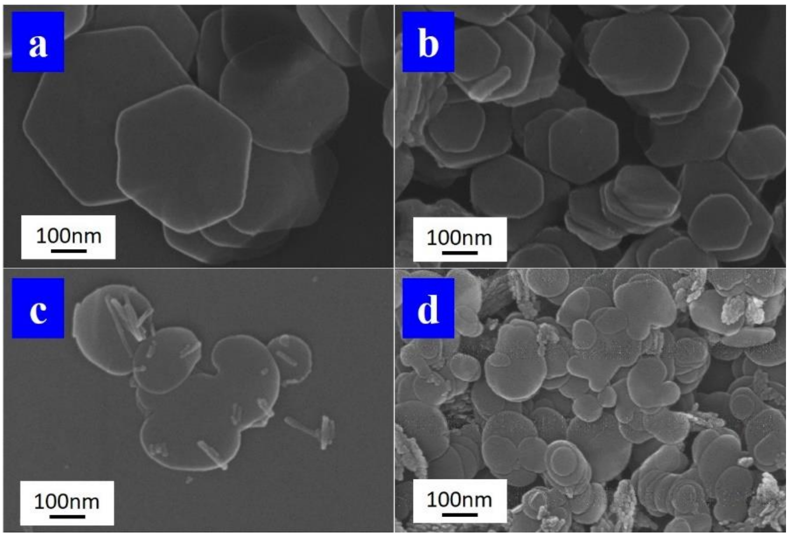


**Supplementary Figure 1.** Morphology of $Cu_xFe_yMg_4$-LDH. SEM images of (a) $Cu_1Fe_1Mg_4$-LDH, (b) $Cu_2Fe_1Mg_4$-LDH, (c) $Cu_4Fe_1Mg_4$-LDH and (d) $Cu_6Fe_1Mg_4$-LDH.

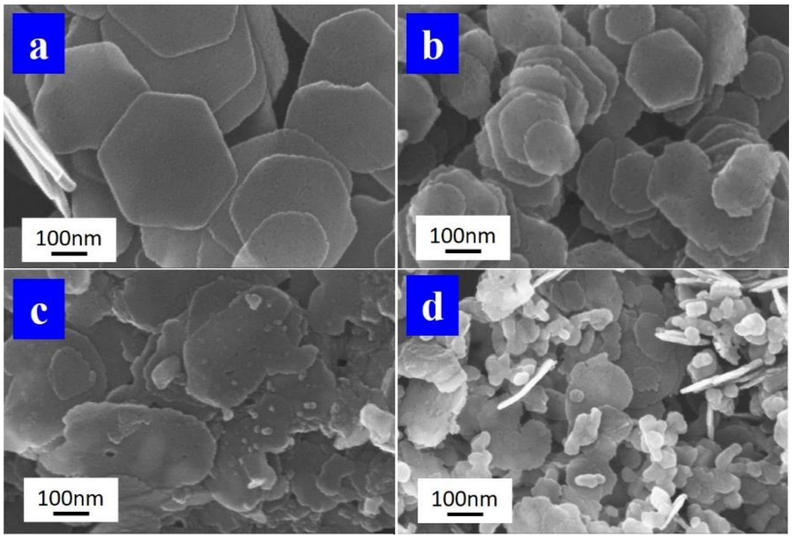


**Supplementary Figure 2.** Morphology of $Cu_xFe_yMg_4$-MMO. SEM images of Calcined sample: (a) $Cu_1Fe_1Mg_4$-MMO, (b) $Cu_2Fe_1Mg_4$-MMO, (c) $Cu_4Fe_1Mg_4$-MMO and (d) $Cu_6Fe_1Mg_4$-MMO.

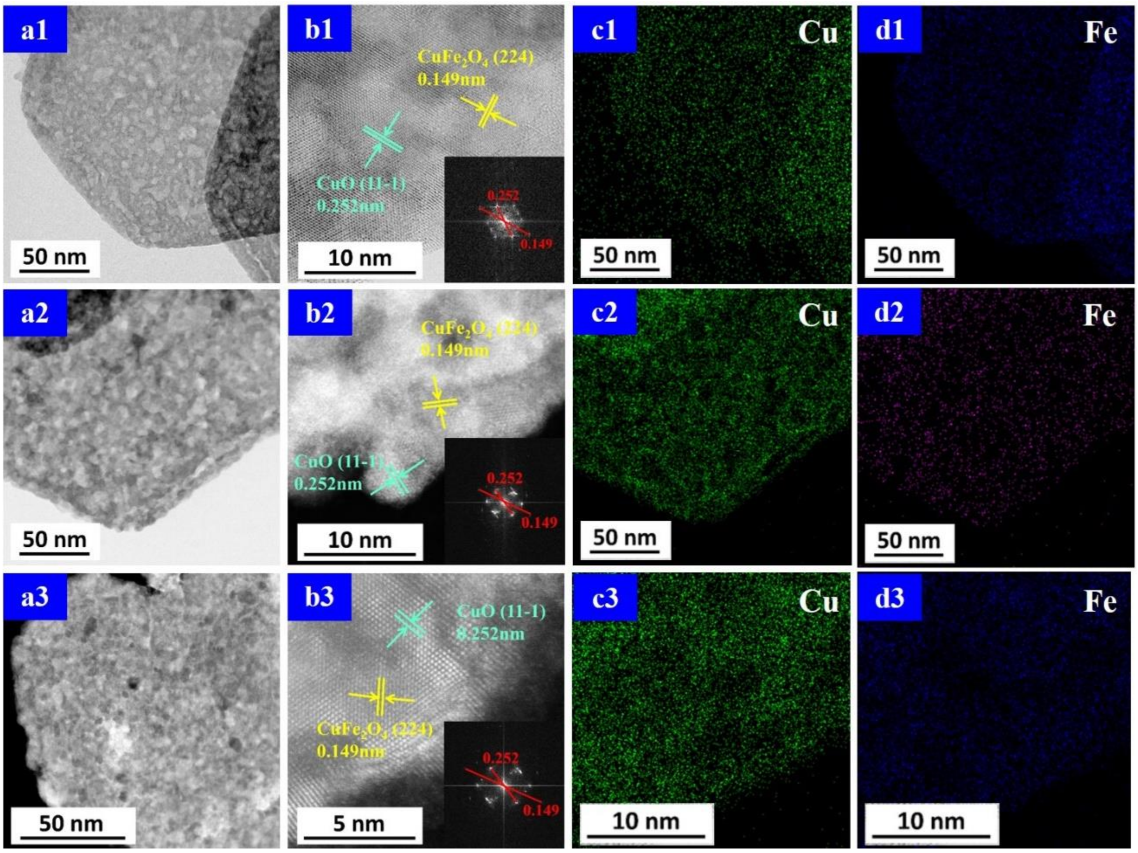


**Supplementary Figure 3.** Morphology of $Cu_xFe_yMg_4$-MMO. TEM images of Calcined sample: (a1–a3) $Cu_1Fe_1Mg_4$-MMO, $Cu_2Fe_1Mg_4$-MMO and $Cu_4Fe_1Mg_4$-MMO. (b1–b3) HRTEM images selected from (a1–a3) and their Fourier transform patterns of the selected region (inset), respectively. (c1–c3) and (d1–d3) EDS mapping of elemental distribution for Cu and Fe.

**Supplementary Table 1.** Physicochemical properties of various catalysts

| Sample | BET surface area ($m^2$ $g^{-1}$) | Surface C content (%) | Cu/Fe ratio [a] | Cu/Fe ratio [b] | Cu Crystallite size [c] (nm) | Mean Cu particle size [d] (nm) |
|---|---|---|---|---|---|---|
| $Cu_1Fe_1$ | 32.49 | 33.53 | 0.63 | 0.99 | 15.7 | 13.4 |
| $Cu_2Fe_1$ | 22.07 | 36.39 | 1.24 | 1.96 | 18.4 | 17.9 |
| $Cu_4Fe_1$ | 15.96 | 35.43 | 2.37 | 3.84 | 21.7 | 21.0 |
| $Cu_6Fe_1$ | 10.38 | 29.99 | 4.47 | 5.67 | 25.1 | - |

[a] Cu/Fe ratio was determined by XPS.

[b] Cu/Fe ratio was determined by inductively coupled plasma-atomic emission spectroscopy (ICP-AES).

[c] Crystallite size was determined by XRD with the Scherrer equation.

[d] Mean Cu particle size was determined by TEM images.

**Supplementary Table 2.** Catalytic performances of samples with various calcination temperatures

| Catalysts[a,b,c] | Conv. [%] | Selectivity[mol%] | | | | Alcohols distribution[%][d] | | | | |
|---|---|---|---|---|---|---|---|---|---|---|
| | | $CH_4$ | $C_{2+}H$ | ROH | $CO_2$ | MeOH | EtOH | PrOH | BuOH | $C_{5+}OH$ |
| $Cu_4Fe_1Mg_4$-MMO(800) | 17.3 | 11.3 | 44.7 | 5.2 | 38.8 | 21.3(1.1) | 59.2(3.1) | 12.0(0.6) | 5.7(0.3) | 1.8(0.1) |
| $Cu_4Fe_1Mg_4$-MMO(500) | 48.7 | 2.8 | 48.0 | 15.6 | 33.6 | 6.0(0.9) | 31.7(4.9) | 5.8(0.9) | 2.5(0.4) | 54.0(8.5) |
| $Cu_4Fe_1Mg_4$-MMO(400) | 43.2 | 4.3 | 47.8 | 19.0 | 28.9 | 10.9(2.1) | 43.5(8.3) | 10.6(2.0) | 5.2(1.0) | 30.8(5.6) |
| $Cu_4Fe_1Mg_4$-MMO(350) | 44.9 | 3.1 | 43.4 | 18.8 | 34.7 | 5.9(1.0) | 49.8(9.3) | 9.2(1.7) | 4.3(0.8) | 31.8(6.0) |
| $Cu_4Fe_1Mg_4$-LDH | 42.1 | 9.8 | 50.3 | 18.7 | 21.2 | 26.6(4.9) | 45.3(8.4) | 14.1(2.6) | 6.2(1.2) | 7.8(1.6) |

[a] Activation conditions: 1 g precursor, $H_2$: CO: $CO_2$=1: 1: 2 (40 mL $min^{-1}$), 2 ºC $min^{-1}$, 300 ºC 2 h + 350 ºC 1 h.
[b] Reaction conditions: 3 MPa, 260 ºC, $H_2$/CO=2，2400 mL $g_{cat}^{-1}$ $h^{-1}$.
[c] Various calcination temperatures of $Cu_4Fe_1Mg_4$-LDH in brackets.
[d] Normalized data to $S_{ROH}$, mol% in brackets.

**Supplementary Table 3.** Catalytic performances of samples with various activation steps

| Entry[a,b] | Conv. [%] | Selectivity[mol%] | | | | Alcohols distribution[%][c] | | | | |
|---|---|---|---|---|---|---|---|---|---|---|
| | | $CH_4$ | $C_{2+}H$ | ROH | $CO_2$ | MeOH | EtOH | PrOH | BuOH | $C_{5+}OH$ |
| 500℃ 20h | 4.8 | 5.6 | 41.8 | 45.2 | 7.4 | 85.4(38.6) | 1.9(0.8) | 1.5(0.7) | 0.8(0.4) | 10.4(4.7) |
| 350℃ 2h | 33.1 | 1.7 | 64.2 | 7.2 | 26.9 | 8.4(0.6) | 18.4(1.3) | 7.2(0.5) | 1.3(0.1) | 64.7(4.7) |
| 350℃ 10h ($H_2$:CO:$CO_2$:$N_2$=1:1:2:8, 100 ml min$^{-1}$) | 12.8 | 2.3 | 63.9 | 13.2 | 20.6 | 20.8(2.7) | 25.3(3.3) | 5.4(0.7) | 1.2(0.2) | 47.3(6.3) |
| 300℃ 2h + 350℃ 1h | 48.7 | 2.8 | 48.0 | 15.6 | 33.6 | 6.0(0.9) | 31.7(4.9) | 5.8(0.9) | 2.5(0.4) | 54.0(8.5) |

[a] Activation conditions : $Cu_4Fe_1Mg_4$-MMO, 1 g precursor, $H_2$:CO:$CO_2$=1:1:2 (40 ml min$^{-1}$), 2 ºC min$^{-1}$.
[b] Reaction conditions: 3 MPa, 260 ºC, $H_2$/CO=2，2400 mL $g_{cat}^{-1}$ $h^{-1}$.
[c] Normalized data to $S_{ROH}$, mol% in brackets.

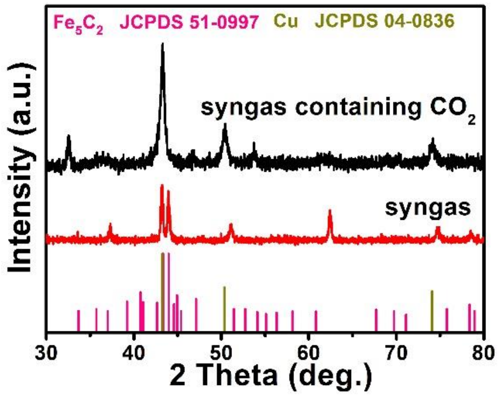


**Supplementary Figure 4.** XRD patterns of the reduced and passivated samples in syngas or syngas containing $CO_2$ of $Cu_4Fe_1Mg_4$-MMO.

**Supplementary Table 4.** Catalytic performances of samples with various activation atmosphere

| Activation atmosphere[a,b] | Pressure [MPa] | Conv. [%] | Selectivity[mol%] | | | | Alcohols distribution[%][c] | | | | |
|---|---|---|---|---|---|---|---|---|---|---|---|
| | | | $CH_4$ | $C_{2+}H$ | ROH | $CO_2$ | MeOH | EtOH | PrOH | BuOH | $C_{5+}OH$ |
| 50.0% $CO_2$ | 1 | 53.2 | 3.7 | 36.5 | 29.8 | 30.0 | 8.7(2.6) | 28.8(8.5) | 8.2(2.4) | 5.2(1.5) | 49.1(14.8) |
| | 2 | 39.9 | 2.8 | 60.3 | 17.9 | 19.4 | 10.4(1.8) | 41.7(7.4) | 9.9(1.7) | 9.1(1.6) | 28.9(5.4) |
| | 3 | 48.7 | 2.8 | 48.0 | 15.6 | 33.6 | 6.0(0.9) | 31.7(4.9) | 5.8(0.9) | 2.5(0.4) | 54.0(8.5) |
| Pure syngas | 1 | 39.3 | 7.6 | 46.6 | 20.7 | 25.1 | 21.6(4.4) | 45.1(9.3) | 14.2(2.9) | 7.3(1.5) | 11.8(2.6) |
| | 2 | 39.7 | 7.8 | 44.8 | 23.0 | 24.4 | 19.2(4.4) | 43.1(9.9) | 13.4(3.1) | 10.2(2.3) | 14.1(3.3) |
| | 3 | 48.9 | 7.8 | 48.7 | 19.2 | 24.3 | 15.0(2.9) | 36.5(7.0) | 10.0(1.9) | 5.2(1.0) | 33.3(6.4) |

[a] Activation conditions: $Cu_4Fe_1Mg_4$-MMO, 1 g precursor, 40 mL $min^{-1}$, 2 ºC $min^{-1}$, 300 ºC 2 h + 350 ºC 1 h.
[b] Reaction conditions: 260 ºC, $H_2/CO=2$，2400 mL $g_{cat}^{-1}$ $h^{-1}$.
[c] Normalized data to $S_{ROH}$, mol% in brackets.

**Supplementary Table 5.** Catalytic performances of samples with various $H_2$/CO ratios in reaction gas

| $H_2$/CO ratio[a,b] | Conv. [%] | Selectivity[mol%] | | | | Alcohols distribution[%][c] | | | | |
|---|---|---|---|---|---|---|---|---|---|---|
| | | $CH_4$ | $C_{2+}H$ | ROH | $CO_2$ | MeOH | EtOH | PrOH | BuOH | $C_{5+}OH$ |
| 1/1 | 26.5 | 4.8 | 34.8 | 12.6 | 47.8 | 12.4(1.6) | 38.3(4.8) | 11.2(1.4) | 11.4(1.4) | 26.7(3.4) |
| 2/1 | 48.7 | 2.8 | 48.0 | 15.6 | 33.6 | 6.0(0.9) | 31.7(4.9) | 5.8(0.9) | 2.5(0.4) | 54.0(8.5) |

[a] Activation conditions: $Cu_4Fe_1Mg_4$-MMO, 1 g precursor, $H_2$: CO: $CO_2$=1: 1: 2 (40 mL min$^{-1}$), 2 ºC min$^{-1}$, 300 ºC 2 h + 350 ºC 1 h.
[b] Reaction conditions: 3 MPa, 260 ºC, 2400 mL $g_{cat}^{-1}$ h$^{-1}$.
[c] Normalized data to $S_{ROH}$, mol% in brackets.

**Supplementary Table 6.** Catalytic performances of samples with various reaction temperatures

| Reaction temperature[a,b] | Conv. [%] | Selectivity[mol%] | | | | Alcohols distribution[%][c] | | | | |
|---|---|---|---|---|---|---|---|---|---|---|
| | | $CH_4$ | $C_{2+}H$ | ROH | $CO_2$ | MeOH | EtOH | PrOH | BuOH | $C_{5+}OH$ |
| 220 | 4.6 | 4.7 | 39.9 | 53.8 | 1.6 | 12.2(6.6) | 16.1(8.7) | 10.7(5.7) | 5.4(2.9) | 55.6(29.9) |
| 260 | 48.7 | 2.8 | 48.0 | 15.6 | 33.6 | 6.0(0.9) | 31.7(4.9) | 5.8(0.9) | 2.5(0.4) | 54.0(8.5) |
| 300 | 55.4 | 7.4 | 57.3 | 13.7 | 21.6 | 80.3(11.0) | 5.0(0.7) | 3.6(0.5) | 1.7(0.2) | 9.4(1.3) |

[a] Activation conditions: $Cu_4Fe_1Mg_4$-MMO, 1 g precursor, $H_2$: CO: $CO_2$=1: 1: 2 (40 mL min$^{-1}$), 2 ºC min$^{-1}$, 300 ºC 2 h + 350 ºC 1 h.
[b] Reaction conditions: 3 MPa, $H_2$/CO=2， 2400 mL $g_{cat}^{-1}$ h$^{-1}$.
[c] Normalized data to $S_{ROH}$, mol% in brackets.

**Supplementary Table 7.** Catalytic performances of samples with various reaction pressure

| Catalysts[a,b] | Pressure [MPa] | Conv. [%] | Selectivity[mol%] | | | | Alcohols distribution [%][d] | | | | |
|---|---|---|---|---|---|---|---|---|---|---|---|
| | | | $CH_4$ | $C_{2+}H$ | ROH [c] | $CO_2$ | MeOH | EtOH | PrOH | BuOH | $C_{5+}OH$ |
| $Cu_1Fe_1$ | 1 | 28.1 | 6.7 | 50.8 | 25.4 (30.6) | 17.1 | 19.0 (4.8) | 38.5 (9.8) | 15.1 (3.8) | 7.3 (1.8) | 20.1 (5.2) |
| | 2 | 33.5 | 3.8 | 59.1 | 21.8 (25.7) | 15.3 | 12.8 (2.8) | 44.1 (9.6) | 14.2 (3.1) | 7.5 (1.6) | 21.4 (4.7) |
| | 3 | 58.2 | 3.3 | 52.3 | 12.9 (18.8) | 31.5 | 6.8 (0.9) | 47.6 (6.1) | 10.1 (1.3) | 4.6 (0.6) | 30.9 (4.0) |
| $Cu_2Fe_1$ | 1 | 28.8 | 2.1 | 44.8 | 23.6 (33.5) | 29.5 | 10.9 (2.6) | 31.0 (7.3) | 13.4 (3.2) | 8.8 (2.1) | 35.9 (8.4) |
| | 2 | 32.1 | 5.8 | 48.4 | 23.0 (29.7) | 22.7 | 20.6 (4.7) | 20.8 (4.8) | 12.4 (2.8) | 7.5 (1.7) | 38.7 (9.0) |
| | 3 | 38.4 | 2.4 | 60.9 | 16.8 (21.0) | 19.9 | 21.4 (3.6) | 21.6 (3.6) | 9.3 (0.9) | 5.3 (0.4) | 43.4 (7.3) |
| $Cu_4Fe_1$ | 1 | 53.2 | 3.7 | 36.5 | 29.8 (42.6) | 30.0 | 8.7 (2.6) | 28.8 (8.5) | 8.2 (2.4) | 5.2 (1.5) | 49.1 (14.8) |
| | 1 [e] | 31.6 | 8.4 | 47.3 | 20.2 (26.6) | 24.1 | 15.1 (3.1) | 22.5 (4.5) | 13.2 (2.6) | 10.6 (2.1) | 38.6 (7.9) |
| | 2 | 39.9 | 2.8 | 60.3 | 17.9 (22.2) | 19.4 | 10.4 (1.8) | 41.7 (7.4) | 9.9 (1.7) | 9.1 (1.6) | 28.9 (5.4) |
| | 3 | 48.7 | 2.8 | 48.0 | 15.6 (23.5) | 33.6 | 6.0 (0.9) | 31.7 (4.9) | 5.8 (0.9) | 2.5 (0.4) | 54.0 (8.5) |
| $Cu_6Fe_1$ | 1 | 17.2 | 5.7 | 43.6 | 7.1 (12.6) | 43.6 | 4.1(0.3) | 14.7(1.0) | 3.9(0.3) | 2.1(0.1) | 75.2(5.4) |
| | 1 [f] | 30.5 | 6.4 | 46.3 | 3.2 (5.7) | 44.1 | 10.1 (0.3) | 15.5 (0.5) | 9.2 (0.3) | 5.6 (0.2) | 59.6 (1.9) |
| | 2 | 27.5 | 3.1 | 45.2 | 9.8 (16.8) | 41.9 | 9.1(0.9) | 19.8(1.9) | 6.8(0.6) | 3.5(0.3) | 60.8(6.1) |
| | 3 | 61.5 | 10.7 | 38.0 | 8.9 (15.4) | 42.4 | 9.7(0.9) | 29.6(2.6) | 8.2(0.7) | 4.2(0.3) | 48.3(4.4) |

[a] Activation conditions: 1 g precursor, $H_2$: CO: $CO_2$=1: 1: 2 (40 mL $min^{-1}$), 2 ºC $min^{-1}$, 300 ºC 2 h + 350 ºC 1 h.
[b] Reaction conditions : 260 ºC, $H_2$/CO=2, 2400 mL $g_{cat}^{-1}$ $h^{-1}$.
[c] $S_{ROH}$ excluding $CO_2$ in brackets.
[d] Normalized data to $S_{ROH}$, mol% in brackets.
[e] WHSV = 4800 mL $g_{cat}^{-1}$ $h^{-1}$
[f] WHSV = 1200 mL $g_{cat}^{-1}$ $h^{-1}$

**Supplementary Table 8.** Space time yield of various products calculated from Table S7

| Catalysts | Pressure [MPa] | Space time yield [g $g_{cat}^{-1}$ $h^{-1}$] | | | | | |
|---|---|---|---|---|---|---|---|
| | | $CH_4$ | $C_{2+}H$ | $CO_2$ | ROH | MeOH | $C_{5+}OH$ |
| $Cu_1Fe_1$ | 1 | 0.017 | 0.116 | 0.122 | 0.130 | 0.025 | 0.026 |
| | 2 | 0.012 | 0.166 | 0.135 | 0.141 | 0.018 | 0.030 |
| | 3 | 0.018 | 0.254 | 0.481 | 0.141 | 0.010 | 0.040 |
| $Cu_2Fe_1$ | 1 | 0.006 | 0.106 | 0.218 | 0.111 | 0.014 | 0.042 |
| | 2 | 0.014 | 0.109 | 0.159 | 0.122 | 0.024 | 0.045 |
| | 3 | 0.009 | 0.200 | 0.205 | 0.131 | 0.027 | 0.053 |
| $Cu_4Fe_1$ | 1 | 0.012 | 0.111 | 0.285 | 0.201 | 0.018 | 0.101 |
| | 2 | 0.010 | 0.205 | 0.207 | 0.140 | 0.014 | 0.040 |
| | 3 | 0.014 | 0.210 | 0.462 | 0.141 | 0.009 | 0.076 |

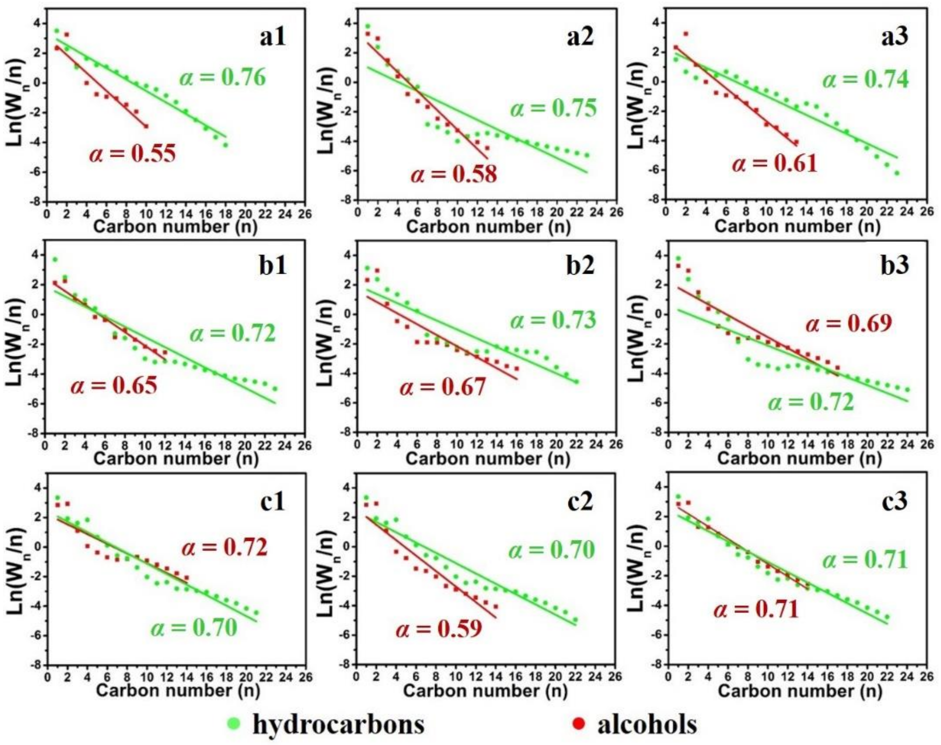


**Supplementary Figure 5.** Anderson-Schulz-Flory (A-S-F) plots for the distribution of alcohols and hydrocarbons. (a) $Cu_1Fe_1$, (b) $Cu_2Fe_1$ and (c) $Cu_4Fe_1$. (a1–c1) at 1 MPa, (a2–c2) at 2 MPa, (a3–c3) at 3 MPa.

**Supplementary Table 9.** Catalytic performance data for a variety of modified F−T catalysts used in LAS

| **Catalyst** | **$H_2$/CO ratio** | **Temperature /℃** | **Pressure /MPa** | **GHSV** | **CO conversion/%** | **Total alcohol Selectivity/%** | **Long-chain alcohol selectivity/%** | **Total alcohols STY[a]** | **Long-chain alcohols STY[a]** | **reference** |
|---|---|---|---|---|---|---|---|---|---|---|
| **$Cu_4Fe_1$** | 2 | 260 | 1 | 2400 mL $g_{cat}^{-1}$ $h^{-1}$ | 53.2 | 29.8 | 49.1 | 0.201 | 0.101 | This work |
| **CuZnFeMn** | 2 | 260 | 4 | 6000 $h^{-1}$ | 52.62 | 31.04 | 3.65 | 0.24 | 0.016 | *Catal. Commun.* **2008**, *9*, 1869-1873 |
| **3DOM $Cu_2Fe_1$** | 1 | 260 | 4.8 | 2000 $h^{-1}$ | 57.5 | 33.6 | 52.4 | 0.20 | 0.100 | *ChemCatChem* **2014**, *6*, 473-478 |
| **$CF_{0.5}$** | 2 | 260 | 4 | 5000 $h^{-1}$ | 17.99 | 20.77 | 2.5 | 0.05 | 0.001 | *J. Colloid Interface Sci.* **2016**, *470*, 162-171 |
| **CNF-2-0.005** | 1.5 | 240 | 5 | 32000 mL $g_{cat}^{-1}$ $h^{-1}$ | 11 | 39 | | 0.53 | | *ACS Catal.* **2018**, *8*, 9604-9618 |
| **CuFe NPs** | 2 | 220 | 6 | 6000 $h^{-1}$ | 17.1 | 21.9 | 64 | 0.14 | 0.085 | *J. Mol. Catal. A: Chem.* **2013**, *378*, 319-325 |
| **0.5%K-FeCuMnZnO** | 2 | 260 | 4 | 6000 $h^{-1}$ | 27.3 | 49.3 | 3 | 0.29 | | *Appl. Energy* **2015**, *138*, 584-589 |
| **CoGa-ZnAl-LDO/ $Al_2O_3$** | 2 | 260 | 3 | 2000 $h^{-1}$ | 43.5 | 59 | 37.7 | 0.24 | 0.091 | *J. Catal.* **2016**, *340*, 236-247 |
| **CoMn \| CuZnAlZr** | 2 | 230 | 6 | 2000 mL $g_{cat}^{-1}$ $h^{-1}$ | 17.8 | 46.1 | 57.1 | 0.04 | 0.023 | *Angew. Chem., Int. Ed.* **2019**, *58, 4627-4631* |
| **CoCu/$MoO_x$** | 1 | 270 | 4 | 120000 mL $g_{cat}^{-1}$ $h^{-1}$ | <2 | 46 | | 0.03 | | *Angew. Chem., Int. Ed.* **2014**, *53*, 6397-6401 |
| **$(Cu_1Co_2)_2$Al/CNT** | 2 | 230 | 3 | 3900 mL $g_{cat}^{-1}$ $h^{-1}$ | 45 | 62 | 13.8 | 0.34 | 0.05 | *J. Mater. Sci.* **2016**, *51*, 5216-5231 |
| **CuFeCo** | 2 | 350 | 5.5 | 6000 $h^{-1}$ | 72 | 12.5 | 6 | 0.25 | 0.015 | *Appl. Catal. A* **2015**, *503*, 51-61 |

| | | | | | | | | | | |
|---|---|---|---|---|---|---|---|---|---|---|
| **Fe/K/ $Mo_2C$(Fe/Mo= 1/14)** | 2 | 320 | 7 | 4000 $h^{-1}$ | 50.25 | 22.69 | 0.62 | 0.14 | 0.001 | *Catal. Lett.* **2010**, *136*, 9-13 |
| **S2-CuFeMg-Cat** | 2 | 300 | 4 | 2000 $h^{-1}$ | 56.89 | 49.07 | 11.25 | 0.28 | 0.032 | *Catal. Sci. Technol.* **2013**, *3*, 1324-1332 |
| **CuFeK0.5M** | 2 | 320 | 5 | 6000 $h^{-1}$ | 53 | 61 | | 0.32 | | *Fuel Process. Technol.* **2017**, *159*, 436-441 |
| **Fe-CuMnZr$O_2$(I)** | 2 | 310 | 8 | 8000 $h^{-1}$ | 45.5 | 26.2 | 3 | 0.45 | 0.014 | *J. Mol. Catal. A: Chem.* **2004**, *221*, 51-58 |
| **Fe-Cu/$Al_2O_3$ ($Al_2O_3$ loading: 89.3%)** | 2.68 | 380 | 4 | 10000 $h^{-1}$ | | 62.3 | | 0.044 | | *J. Nat. Gas Chem.* **2008**, *17*, 327-331 |
| **Cu-Fe-K-$M_{80}$** | 2 | 320 | 5 | 6000 $h^{-1}$ | 56 | 63 | | 0.35 | | *Energy Procedia* **2015**, *75*, 767-772 |
| **$Cu_{20}Fe_{30}K_1M$** | 2 | 320 | 5 | 6000 $h^{-1}$ | 46 | 53 | | 0.35 | | *Catal. Today* **2014**, *234*, 278-284 |
| **K–$CoMoS_2/Al_2O_3$** | 2 | 300 | 5 | 5000 $h^{-1}$ | 9 | 47 | 47 | 0.30 | 0.141 | *Energy Fuels* **2013,** *277*, 3769-3777 |
| **$Co_4Mn_1K_{0.1}$** | 5 | 220 | 4 | 3600 $h^{-1}$ | 34 | 44 | 22 | 0.20 | 0.044 | *Nat Commun.* **2016**, *7*, 13058-13064 |
| **CoCuMn** | 2 | 200 | 6 | 3600 $h^{-1}$ | 3 | 52 | 65 | 0.12 | 0.078 | *J. Am. Chem. Soc.* **2013**, *135*, 7114-7117 |
| **$Co_1Mo_1K_{0.05-12\%}$ (4.2% Co/MWCNT)** | 2 | 290 | 5 | 8000 mL $g_{cat}^{-1}$ $h^{-1}$ | 21.1 | 85 | | 0.33 | | *Appl. Catal. A* **2008**, *340*, 87-97 |

[a] **g $g_{cat}^{-1}$ $h^{-1}$**

**Supplementary Table 10.** Catalytic performances of $Cu_4Fe_1$ with three runs

| Entry[a,b] | Conv. [%] | Selectivity[mol%] | | | | Alcohols distribution[%][c] | | | | |
|---|---|---|---|---|---|---|---|---|---|---|
| | | $CH_4$ | $C_{2+}H$ | ROH | $CO_2$ | MeOH | EtOH | PrOH | BuOH | $C_{5+}OH$ |
| 1 | 53.2 | 3.7 | 36.5 | 29.8 | 30.0 | 8.7(2.6) | 28.8(8.5) | 8.2(2.4) | 5.2(1.5) | 49.1(14.8) |
| 2 | 50.1 | 3.4 | 34.3 | 31.2 | 30.1 | 9.6(3.0) | 29.6(9.2) | 8.7(2.7) | 6.4(1.9) | 45.7(14.4) |
| 3 | 51.2 | 2.3 | 38.6 | 28.0 | 31.1 | 7.5(2.1) | 28.0(7.8) | 8.1(2.3) | 6.0(1.7) | 50.4(14.1) |

[a] Activation conditions: 1 g precursor, $H_2$: CO: $CO_2$=1: 1: 2 (40 mL $min^{-1}$), 2 ºC $min^{-1}$, 300 ºC 2 h + 350 ºC 1 h.
[b] Reaction conditions: 1 MPa, 260 ºC, $H_2$/CO=2, 2400 mL $g_{cat}^{-1}$ $h^{-1}$.
[c] Normalized data to $S_{ROH}$, mol% in brackets.

**Supplementary Table 11.** Catalytic performances of samples with different preparation methods and composition

| Catalysts[a,b] | Conv. [%] | Selectivity[mol%] | | | | Alcohols distribution[%][c] | | | | |
|---|---|---|---|---|---|---|---|---|---|---|
| | | $CH_4$ | $C_{2+}H$ | ROH | $CO_2$ | MeOH | EtOH | PrOH | BuOH | $C_{5+}OH$ |
| $Cu_4Fe_1$ | 53.2 | 3.7 | 36.5 | 29.8 | 30.0 | 8.7(2.6) | 28.8(8.5) | 8.2(2.4) | 5.2(1.5) | 49.1(14.8) |
| $Cu_4Fe_1$-co | 37.1 | 13.4 | 31.3 | 35.2 | 20.1 | 9.6(3.4) | 79.6(28.0) | 6.7(2.3) | 2.4(0.8) | 1.7(0.7) |
| $Cu_4Fe_1$-im | 9.7 | 19.9 | 52.0 | 10.5 | 17.6 | 81.2(8.5) | 9.6(1.0) | 5.3(0.6) | 3.9(0.4) | – |
| $Cu_4$ | 5.9 | 23.1 | 2.1 | 70.5 | 4.3 | 92.0(64.8) | 3.9(2.7) | 2.7(1.9) | 1.4(1.1) | – |
| $Fe_1$ | 19.2 | 21.3 | 38.6 | – | 40.1 | – | – | – | – | – |
| $Cu_4/Fe_1$[d] | 7.8 | 25.5 | 21.7 | 27.6 | 25.2 | 85.6(23.6) | 8.8(2.4) | 5.6(1.6) | – | – |
| $Cu_4+Fe_1$[e] | 6.2 | 21.7 | 47.8 | 15.0 | 15.5 | 89.1(13.3) | 7.3(1.1) | 3.6(0.6) | | |
| $Fe_1+Cu_4$[f] | 6.5 | 27.3 | 34.3 | 19.6 | 18.8 | 90.6(17.7) | 7.6(1.5) | 1.8(0.4) | | |

[a] Activation conditions: 1 g precursor, $H_2$: CO: $CO_2$=1: 1: 2 (40 mL min$^{-1}$), 2 ºC min$^{-1}$, 300 ºC 2 h + 350 ºC 1 h.
[b] Reaction conditions: 1 MPa, 260 ºC, $H_2$/CO=2, 2400 mL $g_{cat}^{-1}$ h$^{-1}$.
[c] Normalized data to $S_{ROH}$, mol% in brackets.
[d] Physical mixture of $Cu_4$ and $Fe_1$
[e] $Fe_1$ is located separately below $Cu_4$ by an inert layer of quartz wool.
[f] $Cu_4$ is located separately below $Fe_1$ by an inert layer of quartz wool.

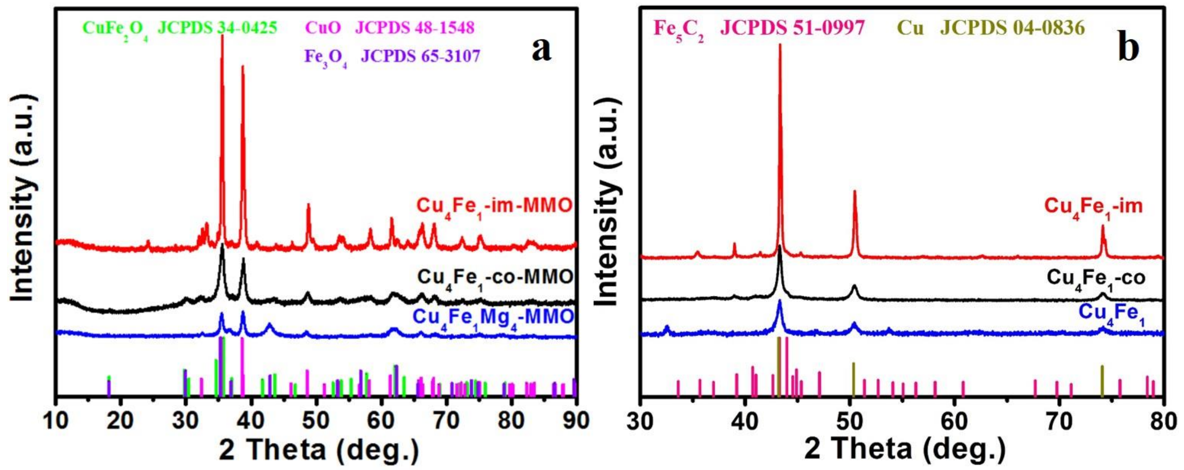


**Supplementary Figure 6.** Structure of control samples. XRD patterns of the calcined samples (a) $Cu_4Fe_1$-im-MMO, $Cu_4Fe_1$-co-MMO, $Cu_4Fe_1Mg_4$-MMO and the reduced samples (b) $Cu_4Fe_1$-im, $Cu_4Fe_1$-co, $Cu_4Fe_1$.

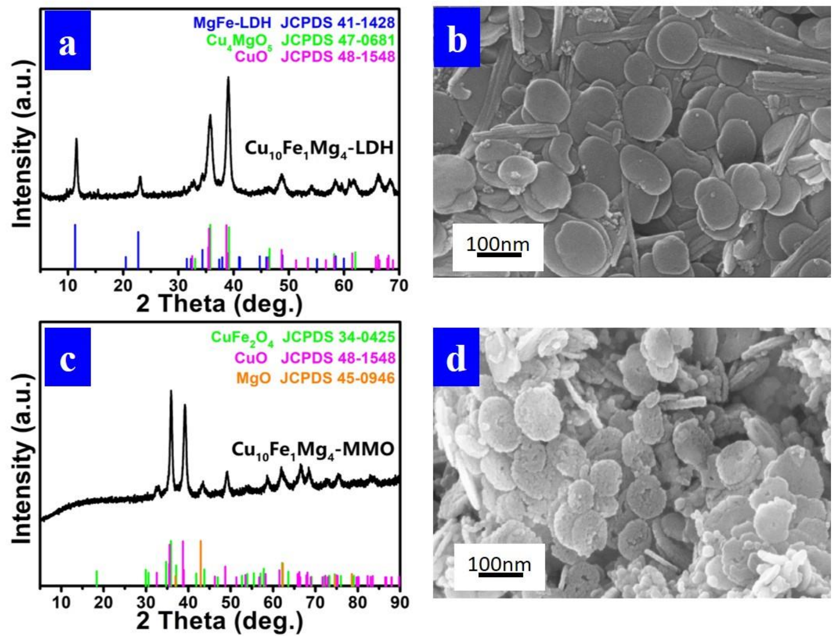


**Supplementary Figure 7.** Structure and morphology of $Cu_{10}Fe_1Mg_4$-LDH. XRD patterns of (a) $Cu_{10}Fe_1Mg_4$-LDH precursors and calcined (c) $Cu_{10}Fe_1Mg_4$-MMO precursors. SEM images: (b) $Cu_{10}Fe_1Mg_4$-LDH and (d) $Cu_{10}Fe_1Mg_4$-MMO.

**Supplementary Table 12.** Catalytic performances of samples with Cu/Fe ratio 10/1

| Catalysts[a,b] | Pressure [MPa] | Conv. [%] | Selectivity[mol%] | | | | Alcohols distribution[%][c] | | | | |
|---|---|---|---|---|---|---|---|---|---|---|---|
| | | | $CH_4$ | $C_{2+}H$ | ROH | $CO_2$ | MeOH | EtOH | PrOH | BuOH | $C_{5+}OH$ |
| $Cu_{10}Fe_1$ | 1 | 17.4 | 9.7 | 41.1 | 5.4 | 43.8 | 11.1(0.6) | 21.2(1.1) | 6.3(0.3) | 3.2(0.2) | 58.2(3.2) |
| | 2 | 32.4 | 5.8 | 45.0 | 10.2 | 39.0 | 12.9(1.3) | 25.5(2.6) | 7.2(0.7) | 3.6(0.4) | 50.8(5.2) |
| | 3 | 61.2 | 15.2 | 26.9 | 10.1 | 47.8 | 11.1(1.1) | 24.0(2.4) | 7.4(0.7) | 4.1(0.4) | 53.4(5.5) |

[a] Activation conditions: 1 g precursor, $H_2$: CO: $CO_2$=1: 1: 2 (40 mL $min^{-1}$), 2 ºC $min^{-1}$, 300 ºC 2 h + 350 ºC 1 h.
[b] Reaction conditions: 260 ºC, $H_2$/CO=2, 2400 mL $g_{cat}^{-1}$ $h^{-1}$.
[c] Normalized data to $S_{ROH}$, mol% in brackets.

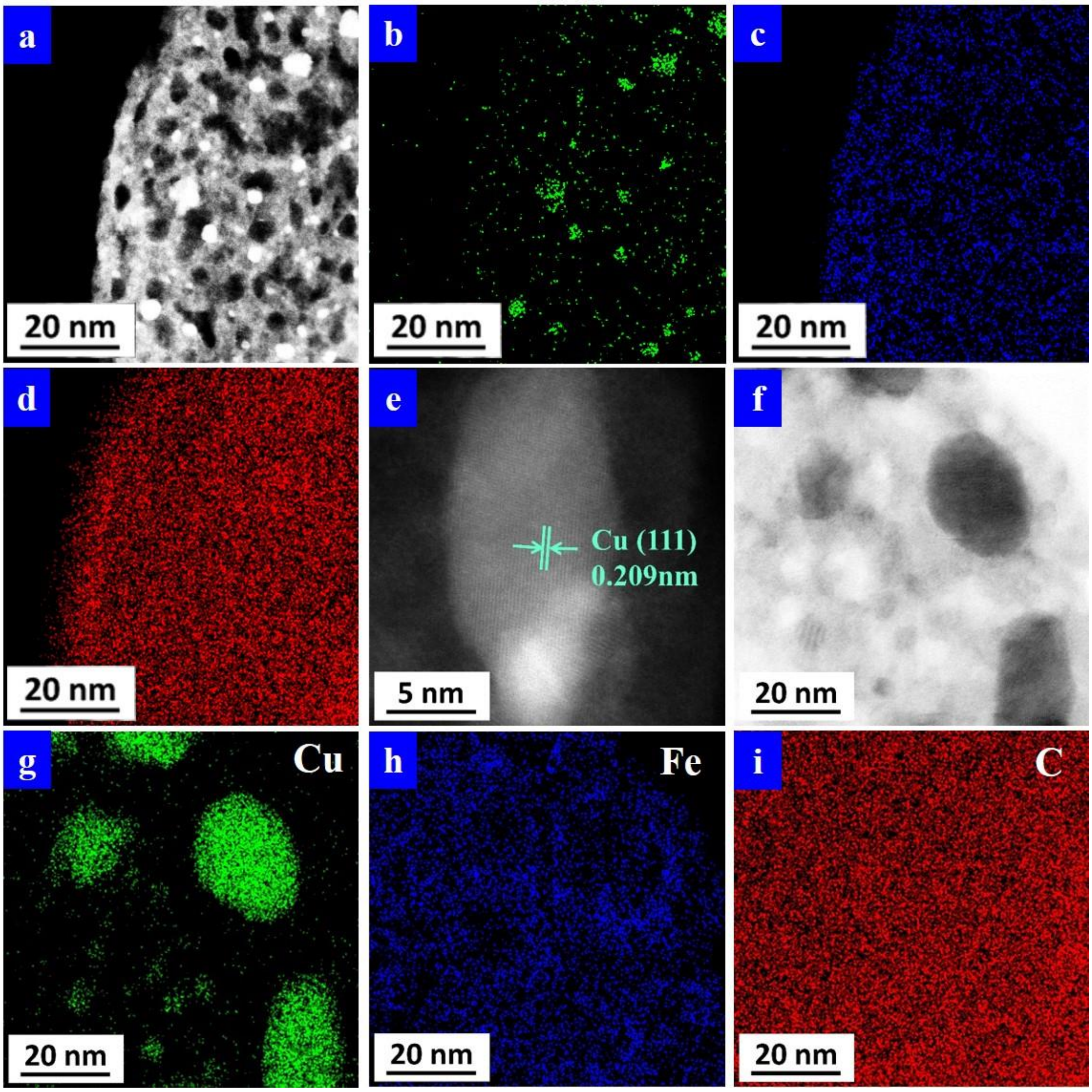


**Supplementary Figure 8.** Morphology of $Cu_1Fe_1$ catalyst. (a, e and f) TEM images of $Cu_1Fe_1$ catalyst (reduced and passivated). (b, c, d, g, h and i) EDS mapping of elemental distribution for Cu, Fe and C, respectively.

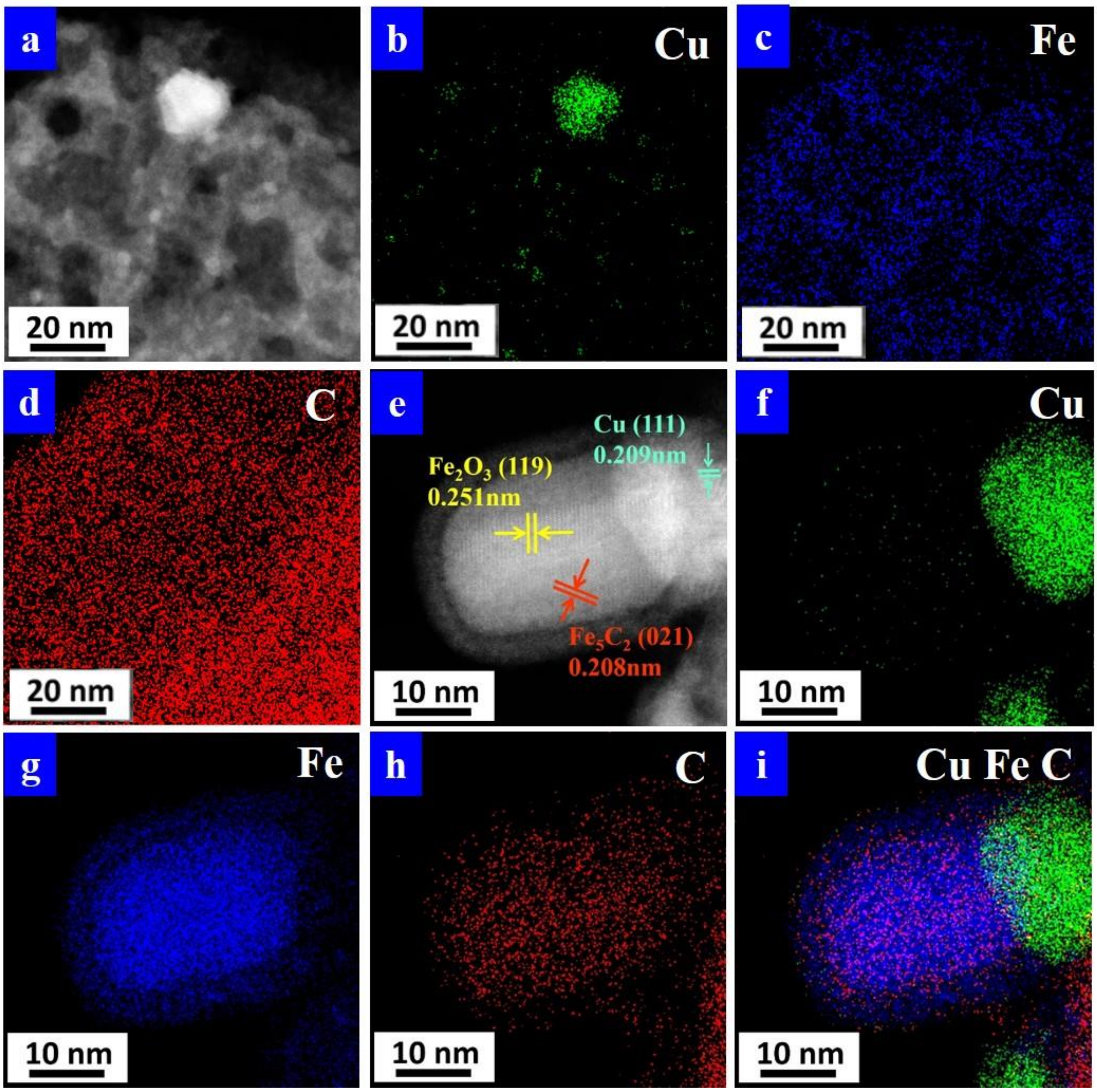


**Supplementary Figure 9.** Morphology of $Cu_2Fe_1$ catalyst. (a, e) TEM images of $Cu_2Fe_1$ catalyst (reduced and passivated). (b, c, d, f, g, h and i) EDS mapping of elemental distribution for Cu, Fe and C, respectively.

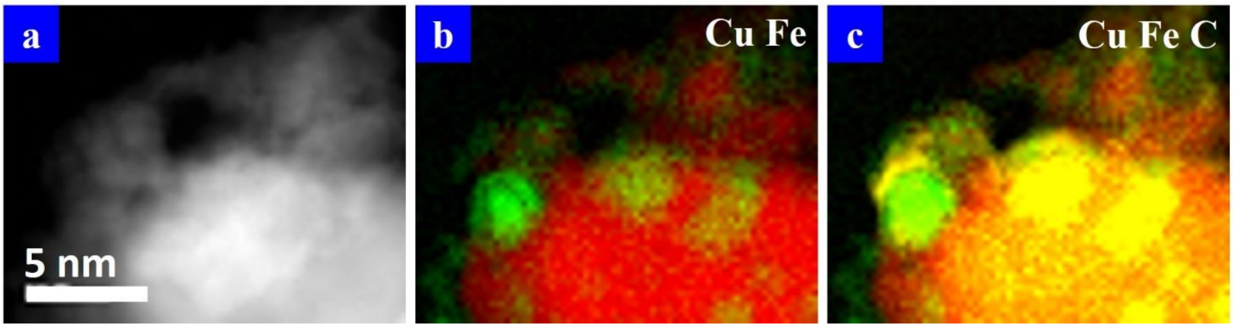


**Supplementary Figure 10.** Morphology of $Cu_4Fe_1$ catalyst. (a) HAADF-STEM image and (b, c) corresponding EELS mapping of Cu (red), Fe (green) and C (yellow).

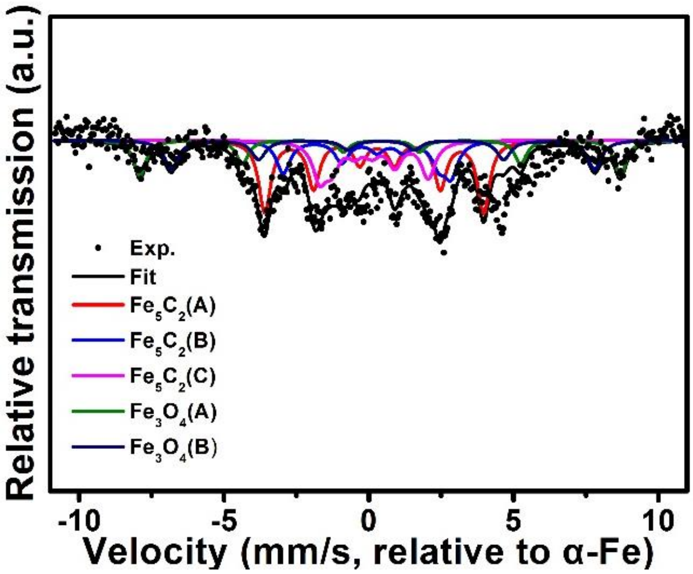


**Supplementary Figure 11.** Mössbauer spectrum of $Cu_4Fe_1$ sample (reduced and passivated) (Exp.: experimental data; Fit: fitting data).

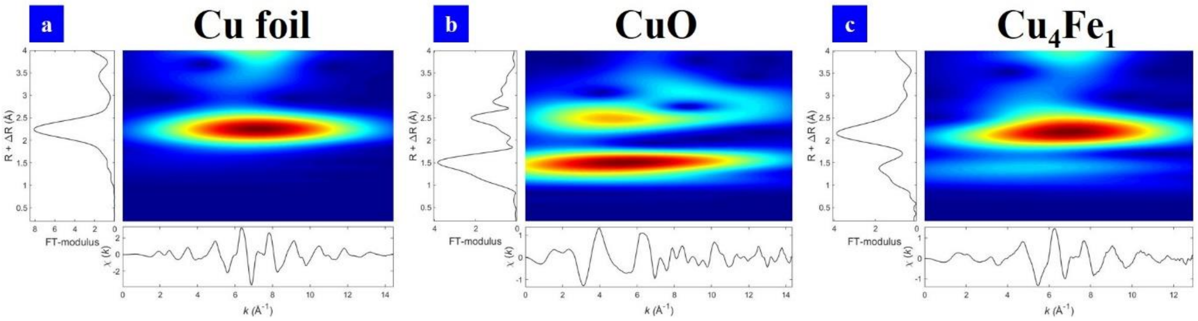


| Sample | Cu-Cu | | | | Cu-O | | | |
|---|---|---|---|---|---|---|---|---|
| | C.N. | $\sigma^2$/*$10^{-3}$(Å$^2$) | $E_0$/eV | R/Å | C.N. | $\sigma^2$/*$10^{-3}$(Å$^2$) | $E_0$/eV | R/Å |
| Cu foil | 12.0 | 8.5 | 4.38 | 2.544±0.002 | - | - | - | - |
| CuO | - | - | - | - | 4.0 | 3.4 | -0.68 | 1.950±0.004 |
| $Cu_4Fe_1$ | 4.6 | 5.2 | -7.13 | 2.46±0.06 | 2.6 | 1.1 | -7.13 | 1.82±0.03 |

**Supplementary Figure 12.** Structure of $Cu_1Fe_1$ catalyst. Wavelet transformation of (a) Cu foil, (b) CuO and (c) $Cu_4Fe_1$. The fitting results are listed in the table.

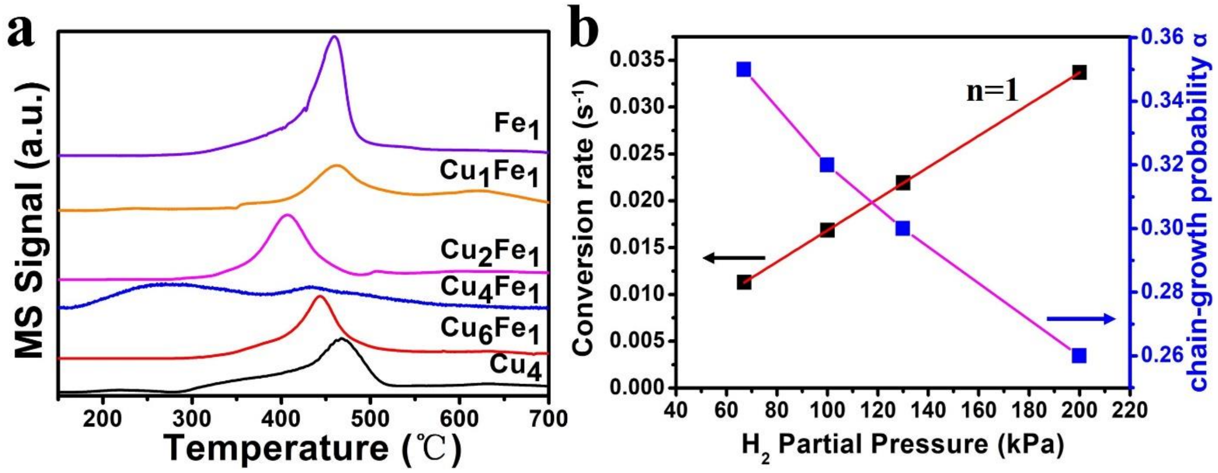


**Supplementary Figure 13.** $H_2$ activation of $Fe_1$, $Cu_4$ and $Cu_xFe_y$ samples. (a) $H_2$-TPD profiles of $Fe_1$, $Cu_4$ and $Cu_xFe_y$ samples. (b) Kinetic orders of $H_2$ and the corresponding chain-growth probability at various $H_2$ partial pressures over $Cu_4Fe_1$.
