# Supplementary material for: Interfacial Fe5C2-Cu catalysts toward low-pressure syngas conversion to long-chain alcohols
Source: Nat Commun. 2020 Jan 3;11:61. doi: 10.1038/s41467-019-13691-4 (PMC6941981; doi:10.1038/s41467-019-13691-4)
Supplement: Supplementary file 2 — Peer Review [file 41467_2019_13691_MOESM2_ESM.pdf]

Reviewers' comments:

Reviewer #1 (Remarks to the Author):

The article reports a CuFe catalyst showing appreciable performance in the synthesis of long-chain alcohols from syngas at mild pressure. Although introducing some interesting results, the quality of this manuscript does not meet the high standard of Nature Communications as such.
Please find detailed comments below.

Introduction:

Also CoMo and CoCu catalyst can mediate the production of long-chain alcohols. As it is written, it seems that only CuFe catalysts can do that. More importantly, what are structural/electronic differences between CuFe catalysts that produce short-chain alcohols and those that enable the synthesis of longer-chain products in the reported studies? This is extremely important and is also poorly addressed in this contribution. The CuFe 3DOM catalyst referenced effectively has a very similar nanostructure (relative size and placement of Cu and Fe) to that featured by the best catalyst in this work. This should be duly discussed in this study. What about the role of the stacking faults of copper identified as the active sites in CuFe 3DOM?

It would be relevant to specify what effect on the economic and environmental footprint of a perspective process the use of a low pressure would have to better justify the impact of the results. Is there any LCA study available?

The introduction focuses on enhancing the Cu-Fe interface as the key strategy to attain long-chin alcohols, but the situation is not so straight forward. It is expected that, if CO insertion into the growing chain is favored by the improved vicinity of Cu and Fe sites, this would happen too quickly before the chain really grows. The authors must be less simplistic about this point.

Mention to the fact that catalysts with different degree of interface will be produced should be added.

The use of LDHs for preparing higher alcohols synthesis catalysts with superior metal intermixing is known, please add appropriate references.

Catalytic results:

The impact of the activation procedure should be better described since it is so vital to attain a performing catalyst. Why is syngas used? Why is it important to add CO2 in large amounts? How do the nanostructures of catalysts activated in different environments compare?

Figure 2: The selectivity of the catalysts must be compared at iso-conversion. Why is there an activation of the catalyst at the beginning of the run? The color code in b and d is confusing. What data points refer to the secondary y-axis in b and how is the chain-grow probability calculated? This reviewer thinks that the legend higher alcohols in b should be long-chain alcohols.
The authors refer to more or less suitable Cu/Fe ratios, which are bulk ratios and hence not relevant to discuss catalytic properties. XPS analysis should be conducted to access surface metals�much ratios.

Correlating structure with performance:

What does semi-in situ mean?

The best catalyst should be compared with the Cu1Fe1 and Cu6Fe1 catalysts, which behave differently, in an explicit manner based on the same in depth characterization applied to Cu4Fe1, i.e. shifting some data from the SI to the main article and adding relevant characterization.

The negligible adsorption of CO on copper should be substantiated adding the profile of the Cu1 sample to the graph. The attribution of interface and isolated Fe carbide species should be better corroborated. What are the signals below 400°C and above 580°C due to? They have been completely ignored.

The discussion on page 13 offers some interesting points and confirms that considering the interface as the sole factor is simplistic. The intrinsic activities of Cu and Fe and their interaction should be modulated by the availability of reactants to attain the right balance between activity, selectivity and chain-length. Some more discussion and data should be added. The diverse capability to activate H_2 can be studied by H_2-TPD. Besides, kinetic analyses studying the reaction order of H_2 will be helpful. In other words, if the effect of H_2 is so relevant, one should see how the chain length varies with different H_2 contents in the feed at distinct pressures. The authors discuss about the rates of CO insertion and hydrocarbon chain termination. Can they be more specific? The fist typically is the cause of the second. What do they refer to as chain termination?

The discussion implies that less interface can be present when the catalyst is operated at higher pressure, so catalysts such as Cu2Fe1 and Cu1Fe1 should be more performing at higher pressure than at 10 bar. Please address this aspect more in depth.

Reviewer #2 (Remarks to the Author):

In this work, authors prepared Cu-Fe binary catalysts with CuxFeyMg4-LDH as the precursors and tested for the C5+OH productions from syngas. The effects of different parameters (i.e., calcination temperature, activation procedure, Cu/Fe ratio, activation procedure and reaction pressure) on the reaction results were evaluated. Among the examined catalysts, Cu4Fe1 from Cu4Fe1Mg4-LDH showed excellent performance at a very low pressure of 1MPa, comparable to these conventional catalysts operated at least 3MPa. Authors used different characterization techniques to demonstrate their hypothesis that the abundant Fe_5C_2-Cu interfacial sites in Cu4Fe1 contribute to the unique performance. In general, this study is interesting and can be accepted for publication. However, some concerns should be addressed before its formal acceptation.
1: Page 3, line 53-54, the corresponding citation should be added.
2: About the H_2-TPR measurements in Page 4 (line 79-81), authors explained that the enhanced amount $CuFe_2O_4$ with increasing Cu/Fe ratio caused the peak shifting to higher temperature. However, after calculation, the weight percentage of $CuFe_2O_4$ in the resultant products Cu1Fe1Mg4-MMO, Cu2Fe1Mg4-MMO, Cu4Fe1Mg4-MMO was about 37.5%, 30%, 21.3%, respectively. This is contrary to the statement that the increased $CuFe_2O_4$ was observed with increasing Cu/Fe ratio? What's the real reason for the peak shifting?
3: For the catalyst activation, why 50% CO_2 is necessary to get better catalytic performance? According to Supplementary Table 4, catalyst reduction under 50% syngas+ 50% CO_2 brought in good enhancement of CO conversion and alcohols production. What role does it play during the activation procedure?
4: Supplementary Table 3 looks very confusing. I believe the last row should be "300 °C 2h + 350 °C 1h". What's the meaning of 'c' and which entry is it for? Please double check this.
5: For sample Cu1Fe1 and Cu2Fe1, CO conversion is increasing with pressure ascending, as listed in Supplementary Table 7, which is a normal phenomenon. However, CO conversion on Cu4Fe1 reduced with increased pressure. Why is a so big difference between Cu4Fe1 and the other two counterparts?
6: In Page 13, line 216, the author mentioned Supplementary Figure 12, but this figure is not included in the supporting information.

7. Authors made the statement that excellent performance at a very low pressure of 1MPa, which is comparable to these conventional catalysts operated at least 3MPa. Please make it clear that the

comparison is based on similar operation temperature as these conventional catalysts.

Reviewer #3 (Remarks to the Author):

Wei and co-workers have studied CuFe catalysts for syngas conversion to higher alcohols. By starting from an LDH precursor they assure intimate contact between the copper and iron phases. They propose that carbon chains grow on the iron carbide phase while at the copper-iron interphase chain termination via CO insertion takes place. This is a very interesting idea together with reasonable activities and selectivities of the catalysts which in principle could warrant publication in Nature Communications. However, in my opinion several issues have to be worked out before a final decision can be taken.

1. The paper contains many ambiguities. In fact one often has to guess about the meaning of symbols, units and procedures during measurements. Their description of Methods is too concise and I suggest that the make extensive descriptions of their procedures in the Supplementary Information. There are too many examples to cover here so I only mention three below (point 2-4).
2. Selectivities are expressed in % or mol% without an explanation whether it is moles of products or (which is more common) in %C.
3. TEM and many other characterizations are applied without specification of the state of the catalyst. Was it calcined, reduced or reduced plus passivated?
4. What is semi-in-situ STEM? Not described what they have done.
5. In the Abstract the authors should report selectivities to higher alcohols in a precise manner.
6. XRD is not based on 'reflection' but on 'diffraction'. So check the paper to change reflections into diffractions.
7. Scientifically my main concern about this paper is that the authors provide little evidence that the chain termination can only take place on the interface between FeC_x and Cu. I suggest that they vary the distance between pure Cu and Fe phases, e.g. by using physical mixtures of different intimacies of Cu and Fe. We know that in bifunctional catalysis the intimacy is key but that surface diffusion can take place and thereby direct contact between active phases is often not necessary.
8. The TEM work is not too convincing to me. For example in Figure 8 (SI) they focus on a large Cu particle to show that FeC is dispersed on or connected with the large Cu particle. What about the many bright dots in Figure 8-a that suggest many small Cu particles? Are these also in direct contact with FeC?

In conclusion, very interesting study but more rigor is needed in writing and in establishing the Fe-Cu interface as the cause for higher alcohol formation.

Response to Reviewers

Reviewer #1:

Reviewer #1 (Remarks to the Author):

The article reports a CuFe catalyst showing appreciable performance in the synthesis of long-chain alcohols from syngas at mild pressure. Although introducing some interesting results, the quality of this manuscript does not meet the high standard of Nature Communications as such.

Please find detailed comments below.

Introduction:

(1) Also CoMo and CoCu catalyst can mediate the production of long-chain alcohols. As it is written, it seems that only CuFe catalysts can do that. More importantly, what are structural/electronic differences between CuFe catalysts that produce short-chain alcohols and those that enable the synthesis of longer-chain products in the reported studies? This is extremely important and is also poorly addressed in this contribution. The CuFe 3DOM catalyst referenced effectively has a very similar nanostructure (relative size and placement of Cu and Fe) to that featured by the best catalyst in this work. This should be duly discussed in this study. What about the role of the stacking faults of copper identified as the active sites in CuFe 3DOM?

Author reply: We thank the reviewer for the comment. According to this comment, we have cited several papers about CoMo and CoCu catalysts in the introduction section to address this issue. Previous studies (*Nat. Commun.* **2016**, *7*, 13058; *Angew. Chem. Int. Ed.* **2014**, *53*, 6397; *Angew. Chem. Int. Ed.* **2019**, *58*, *4627*) have reported that structural difference of catalysts plays a vital role in controlling the formation of long-chain alcohols. For traditional CuFe catalysts, the separate phase of Cu nanoparticles and Fe nanoparticles normally produces lower alcohols (*ACS Catal.* **2018**, *8*, 9604; *J. Colloid Interface Sci.* **2016**, *470*, 162). Therefore, the key issue to obtain long-chain alcohols is to tune the interfacial structure of CuFe catalysts for balancing the reaction rate of C–C bond propagation and CO insertion. As the reviewer mentioned, the work on CuFe

3DOM catalyst (*ACS Catal.* **2017**, *7*, 5500; *ChemCatChem* **2014**, *6*, 473) rationalized the higher alcohols yield in the presence of Cu nanoparticles involving planar defects and lattice strain, visualized by HRTEM and STEM-EDS mapping. This gives us inspiration that we could immobilize Fe_5C_2 clusters onto the surface of Cu nanoparticles to form a new Fe_5C_2-Cu interfacial catalyst for further promoting the catalytic performance of long-chain alcohols synthesis. We found that this Fe_5C_2-Cu interfacial catalyst exhibits a CO conversion of 53.2%, selectivity of 14.8 mol% (total alcohol: 29.8 mol%) and space time yield of 0.101 g g_{cat}^{-1} h^{-1} for long-chain alcohols, with a surprisingly benign reaction pressure of 1 MPa.

- **p.3, Line 45:** rephrase: "Among various binary catalyst systems (Cu-Fe[1,3,8], Cu-Co[9,10], Co-Mo[11,12]), Cu-Fe binary candidates have attracted considerable attention in the production of long-chain alcohols."

- **p.3, Line 57:** rephrase: "Lu *et al.* recently synthesized a 3DOM FeCu catalyst with atomic steps on the Cu surface involving planar defects and lattice strain, which showed excellent performance toward higher alcohols synthesis[3,16]."

(2) It would be relevant to specify what effect on the economic and environmental footprint of a perspective process the use of a low pressure would have to better justify the impact of the results. Is there any LCA study available?

Author reply: We thank the reviewer for the comment. The low pressure in practical operation gives a significant reduction in pressure drop, energy and facility costs, which takes merits of both environmental and economic benefits (*Appl. Catal. A* **2005**, *281*, 225; *Chem. Commun.* **1968**, 1578). Previous studies on binary catalysts gave a high yield of long-chain alcohol (0.014−0.144 g g_{cat}^{-1} h^{-1}), albeit a relatively harsh reaction condition (3−8 MPa) was required. Hence, our work achieved the purpose of low pressure long-chain alcohols synthesis, which has rarely been reported before.

- **p.3, Line 50:** rephrase: "Especially, the low pressure in practical operation gives a significant reduction in pressure drop, energy and facility costs, which takes merits of both environmental and economic benefits[23,24]."

(3) The introduction focuses on enhancing the Cu-Fe interface as the key strategy to attain long-chain alcohols, but the situation is not so straight forward. It is expected that, if CO insertion into the growing chain is favored by the improved vicinity of Cu and Fe sites, this would happen too quickly before the chain really grows. The authors must be less simplistic about this point.

Author reply: We thank the reviewer for the comment. We have improved this issue in the revised manuscript.

• **p.3, Line 59:** rephrase: "If we can prepare a highly dispersed iron carbide species over Cu, where CO is activated but not dissociated, it is possible to largely enhance the density of interfacial sites. This may render an optimized rate of C–C bond propagation on iron carbide sites and CO insertion on Cu/Fe_5C_2 interfacial sites, and thus a high selectivity toward long-chain alcohols would be achieved."

(4) Mention to the fact that catalysts with different degree of interface will be produced should be added.

Author reply: We thank the reviewer for the comment. We have addressed this issue in the revised manuscript.

• **p.3, Line 53:** rephrase: "To achieve this goal, a precise control over the type, dimension and nature of Cu/Fe interface plays a key role in the production of LAS. Maintaining a high degree of Cu-Fe interface is decisive for shifting the products from hydrocarbons to long-chain alcohols."

(5) The use of LDHs for preparing higher alcohols synthesis catalysts with superior metal intermixing is known, please add appropriate references.

Author reply: We thank the reviewer for the comment. Relevant references have been cited in the revised manuscript.

• **p.3, Line 63:** rephrase: "Layered double hydroxides (LDHs), with unique structure that metal cations are distributed in the hydroxide layers at an atomic level, have attracted extensive attention as catalyst precursors for higher alcohols synthesis[10,15,18,28,29]. In this report, we used CuFe-LDHs as a precursor."

Catalytic results:

(6) The impact of the activation procedure should be better described since it is so vital to attain a performing catalyst. Why is syngas used? Why is it important to add CO_2 in large amounts? How do the nanostructures of catalysts activated in different environments compare?

Author reply: We thank the reviewer for the comment. For the activation process, it has been reported that the formation of iron carbide is mainly affected by temperature and gas composition (*J. Am. Chem. Soc.* **2010**, *132*, 14928; *J. Catal.* **2014**, *317*, 135). In this work, we have systematically tried syngas and syngas containing CO_2, and the results showed CO_2 mixed in syngas could slow down the rate of reduction and carburization of Fe, which would maintain the tiny size of Fe_5C_2 to produce abundant Cu-Fe_5C_2 interface sites. Therefore, we used syngas containing 50% CO_2 for the activation of catalyst.

For comparing the structure of catalysts activated in different environments (syngas or syngas containing CO_2), XRD measurements were performed (Supplementary Figure S4). The sample reduced in syngas shows diffraction peaks of both Cu and Fe_5C_2 phase, which displays rather poor catalytic behavior for the production of LA which might be attributed to the poor dispersion of Fe_5C_2. In contrast, the sample reduced in syngas containing CO_2 shows no obvious diffraction peak for Fe_5C_2, and exhibits Fe_5C_2 clusters (~2 nm) immobilized onto the surface of Cu nanoparticles (~25 nm) according to TEM results (Fig. 3), which gives a superior LA selectivity and yield.

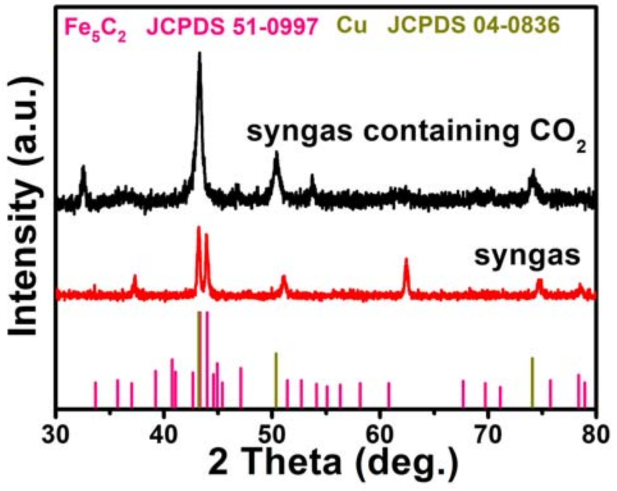

Supplementary Figure S4. XRD patterns of the reduced and passivated $Cu_4Fe_1Mg_4$-MMO in syngas and syngas containing CO_2, respectively.

• **p.6, Line 103:** rephrase: "The sample activated in syngas containing CO_2 (Table S4) could slow down the rate of reduction process and facilitate carburization of Fe to produce Fe_5C_2 species, which would maintain the tiny size of Fe_5C_2 to form abundant Cu-Fe_5C_2 interfacial sites. The sample reduced in syngas shows diffraction peaks of both Cu and Fe_5C_2 phase (Figure S4) and rather poor catalytic behavior for the production of LA, indicating less interfacial sites merged by poor dispersion of Fe_5C_2 is not beneficial for the production of LA."

(7) Figure 2: The selectivity of the catalysts must be compared at iso-conversion. Why is there an activation of the catalyst at the beginning of the run? The color code in b and d is confusing. What data points refer to the secondary y-axis in b and how is the chain-grow probability calculated? This reviewer thinks that the legend higher alcohols in b should be long-chain alcohols.

Author reply: We thank the reviewer for the comment. We have supplemented the catalytic performance data of Cu_4Fe_1 with iso-conversion, in comparison with Cu_1Fe_1 and Cu_2Fe_1 through altering WHSV (see Supplementary Table S7). The catalyst activation at the beginning of the run is due to the carbon deposition on catalyst surface in the reduction process detected by XPS (Supplementary Table S1), which might have slowed the diffusion rate of reactants, and once the carbon wa？ removed by hydrogenation after switching to operating condition, the reaction rate would accelerate. We have improved Figure 2 to give a clear illustration, and described the detailed calculation method of chain-grow probability in the revised manuscript.

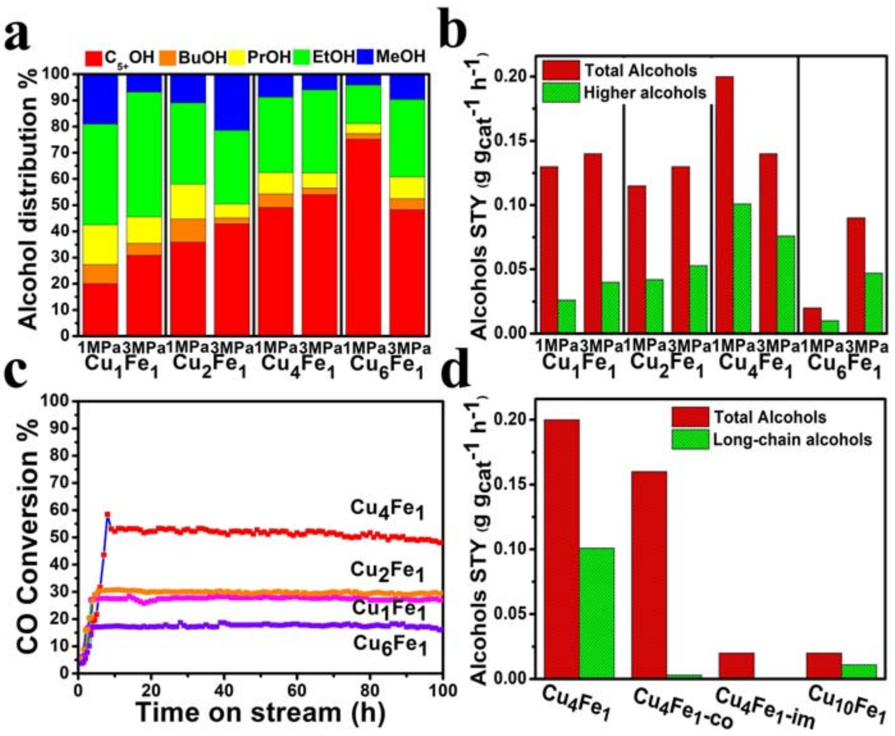

Fig. 2 (a) Alcohol distribution (normalized data to total alcohols selectivity) and (b) alcohols STY at different pressures over catalysts with four Cu/Fe ratios (1/1, 2/1, 4/1 and 6/1) (reaction conditions: 27% CO + 55% H_2 + 18% N_2; 260 °C; WHSV of 2400 mL g_{cat}^{-1} h^{-1}). (c) Time-on-stream (TOS) evolution of CO conversion over the four catalysts within 100 h test at 1 MPa. (d) Alcohols STY of Cu_4Fe_1, Cu_4Fe_1-co, Cu_4Fe_1-im and $Cu_{10}Fe_1$ at 1 MPa.

• **p.18, Line 289:** rephrase: "The ASF chain growth probability α is calculated according to the equation: $\ln (W_n/n) = n\ln\alpha + \ln(1-\alpha)^2/\alpha$, where n is the number of carbon atoms in products; W_n is the weight fraction of products containing n carbon atoms; and $1-\alpha$ is the probability of chain termination."

(8) The authors refer to more or less suitable Cu/Fe ratios, which are bulk ratios and hence not relevant to discuss catalytic properties. XPS analysis should be conducted to access surface metals ratios.

Author reply: We thank the reviewer for the comment. According to this comment, we have performed XPS measurements for Cu_1Fe_1, Cu_2Fe_1, Cu_4Fe_1 and Cu_6Fe_1, and the surface Cu/Fe ratios were listed in Supplementary Table S1.

Supplementary Table S1. Physicochemical properties of various catalysts

Sample	BET surface area (m^2 g^{-1})	Surface C content (%)	Cu/Fe ratio [a]	Cu/Fe ratio [b]	Cu Crystallite size [c] (nm)	Mean Cu particle size [d] (nm)
Cu_1Fe_1	32.49	33.53	0.63	0.99	15.7	13.4
Cu_2Fe_1	22.07	36.39	1.24	1.96	18.4	17.9
Cu_4Fe_1	15.96	35.43	2.37	3.84	21.7	23.0
Cu_6Fe_1	10.38	29.99	4.47	5.67	25.1	-

[a] Cu/Fe ratio was determined by XPS.

[b] Cu/Fe ratio was determined by inductively coupled plasma-atomic emission spectroscopy (ICP-AES).

[c] Crystallite size was determined by XRD with the Scherrer equation.

[d] Mean Cu particle size was determined by TEM images.

Correlating structure with performance:

(9) What does semi-in situ mean?

Author reply: We thank the reviewer for the comment. The sample in this experiment was hold in glovebox in Ar atmosphere and transferred by a vacuum transfer TEM holder. By consulting related descriptions in previous reports, we have changed '*semi-in-situ*' into '*quasi-in-situ*'.

- **p.18, Line 299:** rephrase: "The *quasi-in-situ* scanning transmission electron microscopy (STEM) and EDX mapping measurements were performed on a FEI Tecnai G2 F20 microscope with an accelerating voltage of 120 kV. The sample was hold in glovebox in Ar atmosphere and transferred by a vacuum transfer TEM holder."

(10) The best catalyst should be compared with the Cu1Fe1 and Cu6Fe1 catalysts, which behave differently, in an explicit manner based on the same in depth characterization applied to Cu4Fe1, i.e. shifting some data from the SI to the main article and adding relevant characterization.

Author reply: We thank the reviewer for the comment. According to this comment, we have supplemented experimental data of Cu_6Fe_1 in the revised manuscript and supplementary information.

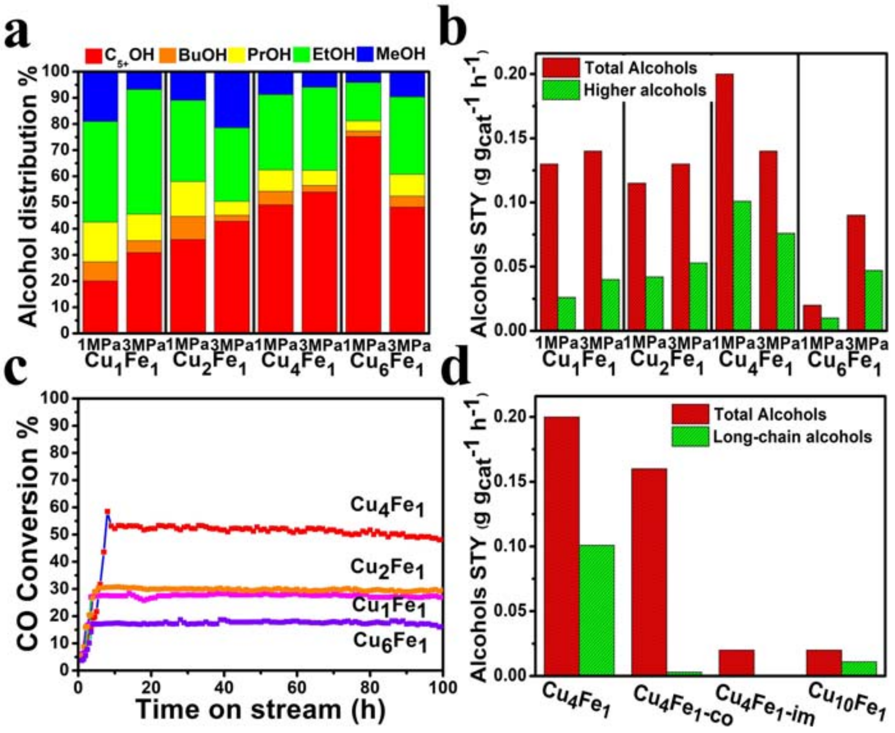

Fig. 2 (a) Alcohol distribution (normalized data to total alcohols selectivity) and (b) alcohols STY at different pressures over catalysts with four Cu/Fe ratios (1/1, 2/1, 4/1 and 6/1) (reaction conditions: 27% CO + 55% H_2 + 18% N_2; 260 °C; WHSV of 2400 mL g_{cat}^{-1} h^{-1}). (c) Time-on-stream (TOS) evolution of CO conversion over the four catalysts within 100 h test at 1 MPa. (d) Alcohols STY of Cu_4Fe_1, Cu_4Fe_1-co, Cu_4Fe_1-im and $Cu_{10}Fe_1$ at 1 MPa.

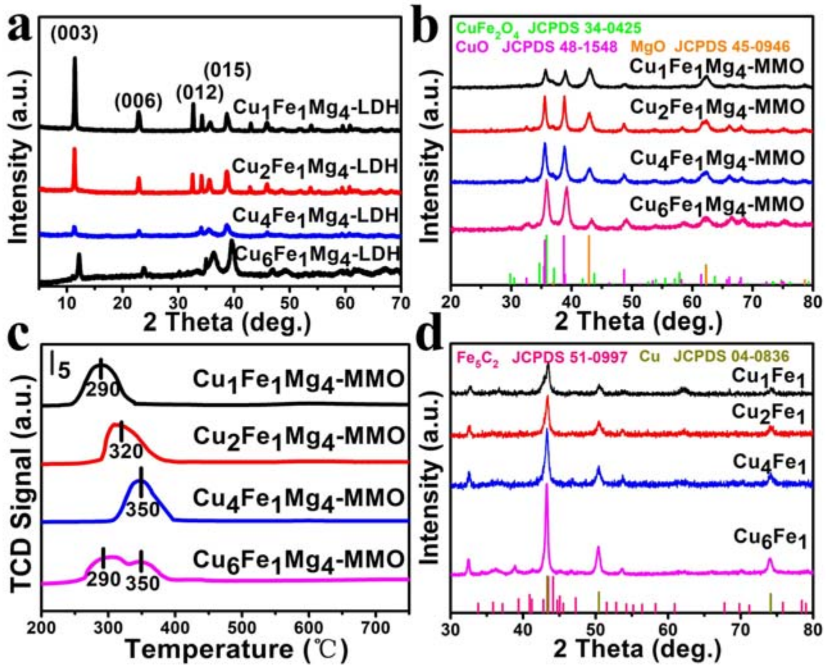

Fig. 1 (a) XRD patterns of $Cu_xFe_yMg_4$-LDH precursors with three molar ratios of Cu/Fe (1:1, 2:1, 4:1 and 6:1, respectively). (b) XRD patterns of $Cu_xFe_yMg_4$-MMO calcined samples obtained from calcination of LDHs precursors. (c) H_2-TPR profiles of $Cu_xFe_yMg_4$-MMO samples. (d) XRD patterns of Cu_xFe_y obtained from activation of MMO samples in syngas (25% CO + 25% H_2 + 50% CO_2) under optimum conditions (300 °C (2 h) + 350 °C (1 h); rate: 2 °C min^{-1}) and passivation.

• **p.4, Line 73:** rephrase: "The $Cu_xFe_yMg_4$-LDH precursors with different Cu/Fe molar ratios (1/1, 2/1, 4/1 and 6/1, respectively) were prepared by a nucleation and aging separation method developed previously[15]. The XRD patterns (Figure 1a) show characteristic diffractions corresponding to an LDH phase (JCPDS 14-0281); SEM images display a typical plate-like hexagonal morphology (Figure S1). Actually, $Cu_6Fe_1Mg_4$-LDH could hardly give an LDH phase due to the strong Jahn-Teller effect of Cu^{2+}."

• **p.5, Line 90:** rephrase: "The Cu_xFe_y catalysts show similar XRD patterns (Figure 1d) with metallic Cu. The average Cu particle sizes are 15.7 nm, 18.4 nm, 21.7 nm and 25.1 nm for Cu_1Fe_1, Cu_2Fe_1, Cu_4Fe_1 and Cu_6Fe_1 samples, respectively (Table S1). Moreover, no obvious

diffraction of Fe crystalline is resolved, which indicates that Fe species is highly dispersed in the catalyst."

(11) The negligible adsorption of CO on copper should be substantiated adding the profile of the Cu1 sample to the graph. The attribution of interface and isolated Fe carbide species should be better corroborated. What are the signals below 400°C and above 580°C due to? They have been completely ignored.

Author reply: We thank the reviewer for the comment. We have supplemented the CO-TPD profile of Cu in Figure 4 and corresponding discussion has been added in the revised manuscript.

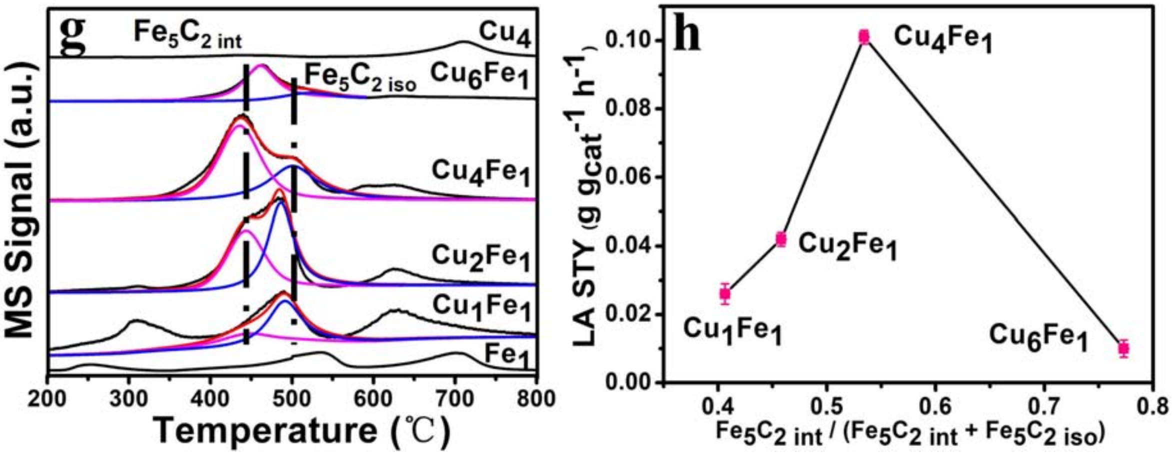

Figure 4. (g) CO-TPD profiles of Fe_1, Cu_4 and Cu_xFe_y samples. (h) Long-chain alcohols space time yield as a function of $Fe_5C_{2int}/(Fe_5C_{2int} + Fe_5C_{2iso})$. Fe_5C_{2int}: interfacial Fe_5C_2; Fe_5C_{2iso}: isolated Fe_5C_2.

• **p.13, Line 199:** rephrase: "As CO adsorption on Cu is rather weak and only be resolved through low temperature TPD[49,50], the observed desorption peaks are ascribed to iron species. In addition to the CO desorption from iron oxide at relatively low temperature (around 300 °C, for Cu_1Fe_1) and reverse Boudouard reaction (above 600 °C), two desorption peaks at 440 °C and 490 °C (for Cu_1Fe_1, Cu_2Fe_1, Cu_4Fe_1 and Cu_6Fe_1) could be attributed to CO desorption from iron carbide[51,52]."

(12) The discussion on page 13 offers some interesting points and confirms that considering the interface as the sole factor is simplistic. The intrinsic activities of Cu and Fe and their

interaction should be modulated by the availability of reactants to attain the right balance between activity, selectivity and chain-length. Some more discussion and data should be added. The diverse capability to activate H2 can be studied by H2-TPD. Besides, kinetic analyses studying the reaction order of H2 will be helpful. In other words, if the effect of H2 is so relevant, one should see how the chain length varies with different H2 contents in the feed at distinct pressures. The authors discuss about the rates of CO insertion and hydrocarbon chain termination. Can they be more specific? The fist typically is the cause of the second. What do they refer to as chain termination?

Author reply: We thank the reviewer for the valuable comment. According to this comment, we have performed H_2-TPD measurements and the data were shown in Supplementary Figure S13a. Compared with other Cu_xFe_y samples, Cu_4Fe_1 gives the weakest activation adsorption of H_2, which means that the hydrogenation of carbon-chain to form hydrocarbons is suppressed. This thus would promote the matching degree of reaction rate between CO dissociation and C–C bond propagation. Hence, Cu_4Fe_1 shows the low-pressure performance for LAS, which is also identified by the result of H_2 kinetic orders (n=1) (Supplementary Figure S13b). The reduction of surface hydrogen concentration not only facilitates CO activation and carbon-chain growth, but also increases the probability of CO insertion. Meanwhile, the selectivity of hydrocarbons and alcohols directly reflects the rate of hydrocarbon chain termination and CO insertion. Cu_4Fe_1 has a weak hydrogenation capacity, which promotes the activation of CO and narrows the rate gap for CO insertion. Therefore, Cu_4Fe_1 gives a high CO conversion and LA yield at 1 MPa.

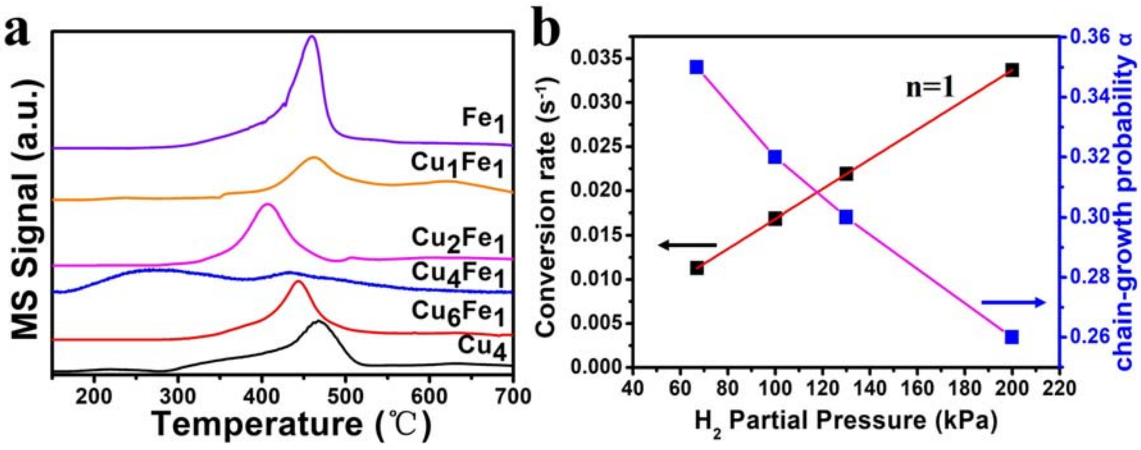

Supplementary Figure S13. (a) H_2-TPD profiles of Fe_1, Cu_4 and Cu_xFe_y samples. (b) Kinetic orders of H_2 and the corresponding chain-growth probability at various H_2 partial pressures.

• **p.14, Line 222:** rephrase: "Compared with other Cu_xFe_y samples, Cu_4Fe_1 shows the weakest ability toward H_2 adsorption and activation (Figure S13a). As the reaction pressure decreases from 3 MPa to 1 MPa, hydrogen activation on catalyst surface is weakened (Figure S13b), which reduces the rate of hydrogenation and hydrocarbons chain termination and thus enhances the growth of carbon-chain. In contrast, the CO activation is promoted since CO molecule is prone to adsorb on a Cu-rich surface[56,57]. This facilitates the kinetic rate matching between CO insertion and C–C coupling, and therefore significantly elevates the selectivity toward total alcohols and long-chain alcohols[58-60]."

(13) The discussion implies that less interface can be present when the catalyst is operated at higher pressure, so catalysts such as Cu2Fe1 and Cu1Fe1 should be more performing at higher pressure than at 10 bar. Please address this aspect more in depth.

Author reply: We thank the reviewer for the comment. In our work, the different interface structures of Cu_1Fe_1, Cu_2Fe_1 and Cu_4Fe_1 are established through activation, demonstrating that the interfacial property is dependent on the composition and activation process of catalysts. Based on the results of this work, we consider that the change of reaction pressure would not affect the catalyst structure but the reaction mechanism. According to this comment, we further discussed this issue in the revised manuscript.

• **p.13, Line 204:** rephrase: "With Fe_1 as reference, these two peaks (440 °C and 490 °C) are attributed to interfacial Fe_5C_2 (Fe_5C_{2int}) and isolated Fe_5C_2 (Fe_5C_{2iso}) species, respectively. Clearly, Cu_6Fe_1 catalyst has the highest density of interfacial Fe_5C_2 sites. We fitted and deconvoluted the two peaks to roughly estimate the ratio of Fe_5C_{2int}/(Fe_5C_{2int}+ Fe_5C_{2iso}), and the results showed a volcanic correlation between the yield of long-chain alcohols and the relative concentration of interfacial Fe_5C_2. Cu_4Fe_1 catalyst possesses the highest concentration of Fe_5C_2-Cu interface sites (Fe_5C_{2int}), accounting for the largest long-chain alcohols yield (0.101 g g_{cat}^{-1} h^{-1}). This demonstrates that the Fe_5C_2–Cu interfacial sites act as active center toward LAS. "

• **p.14, Line 222:** rephrase: "Compared with other Cu_xFe_y samples, Cu_4Fe_1 shows the weakest ability toward H_2 adsorption and activation (Figure S13a). As the reaction pressure decreases from 3 MPa to 1 MPa, hydrogen activation on catalyst surface is weakened (Figure S13b), which reduces the rate of hydrogenation and hydrocarbons chain termination and thus enhances the growth of carbon-chain. In contrast, the CO activation is promoted since CO molecule is prone to adsorb on a Cu-rich surface[56,57]. This facilitates the kinetic rate matching between CO insertion and C−C coupling, and therefore significantly elevates the selectivity toward total alcohols and long-chain alcohols[58-60]. This is also verified by the α-ASF chain-lengthening probabilities analysis: the α-value for alcohols (0.72) at 1 MPa exceeds that for hydrocarbons (0.70), indicating the rate of CO insertion is larger than that of hydrocarbons chain termination (Figure S4). Therefore, a precise control over double-active-site in Cu_4Fe_1 catalyst accounts for the high yield toward long-chain alcohols at 1 MPa."

Reviewer #2:

In this work, authors prepared Cu-Fe binary catalysts with $Cu_xFe_yMg_4$-LDH as the precursors and tested for the $C_{5+}OH$ productions from syngas. The effects of different parameters (i.e., calcination temperature, activation procedure, Cu/Fe ratio, activation procedure and reaction pressure) on the reaction results were evaluated. Among the examined catalysts, Cu4Fe1 from $Cu_4Fe_1Mg_4$-LDH showed excellent performance at a very low pressure of 1MPa, comparable to these conventional catalysts operated at least 3MPa. Authors used different characterization techniques to demonstrate their hypothesis that the abundant Fe_5C_2-Cu interfacial sites in Cu4Fe1 contribute to the unique performance. In general, this study is interesting and can be accepted for publication. However, some concerns should be addressed before its formal acceptation.

(1) Page 3, line 53-54, the corresponding citation should be added.

Author reply: We thank the reviewer for the comment. We have added the corresponding citation in the revised manuscript.

(2) About the H_2-TPR measurements in Page 4 (line 79-81), authors explained that the enhanced amount $CuFe_2O_4$ with increasing Cu/Fe ratio caused the peak shifting to higher temperature. However, after calculation, the weight percentage of $CuFe_2O_4$ in the resultant products $Cu_1Fe_1Mg_4$-MMO, $Cu_2Fe_1Mg_4$-MMO, $Cu_4Fe_1Mg_4$-MMO was about 37.5%, 30%, 21.3%, respectively. This is contrary to the statement that the increased $CuFe_2O_4$ was observed with increasing Cu/Fe ratio? What's the real reason for the peak shifting?

Author reply: We thank the reviewer for the comment. Actually, Cu and Fe could form $CuFe_2O_4$ phase at a high temperature; and CuO phase is predominant for these three samples proved by XRD (Figure 1b). No obvious diffraction peak of iron oxides is detected by XRD; and highly dispersed iron oxide in mixed metal oxides (MMO) is observed in STEM mapping images (Figure S3). This indicates that the amount of $CuFe_2O_4$ could not be calculated by the highest theoretical content. Due to a higher reduction temperature of $CuFe_2O_4$ spinel relative to CuO (*Appl. Catal. A* **2010**, *375*, 163; *Appl. Catal. B* **2007**, *74*, 144), the main temperature peak shifts from 280 °C to 350 °C.

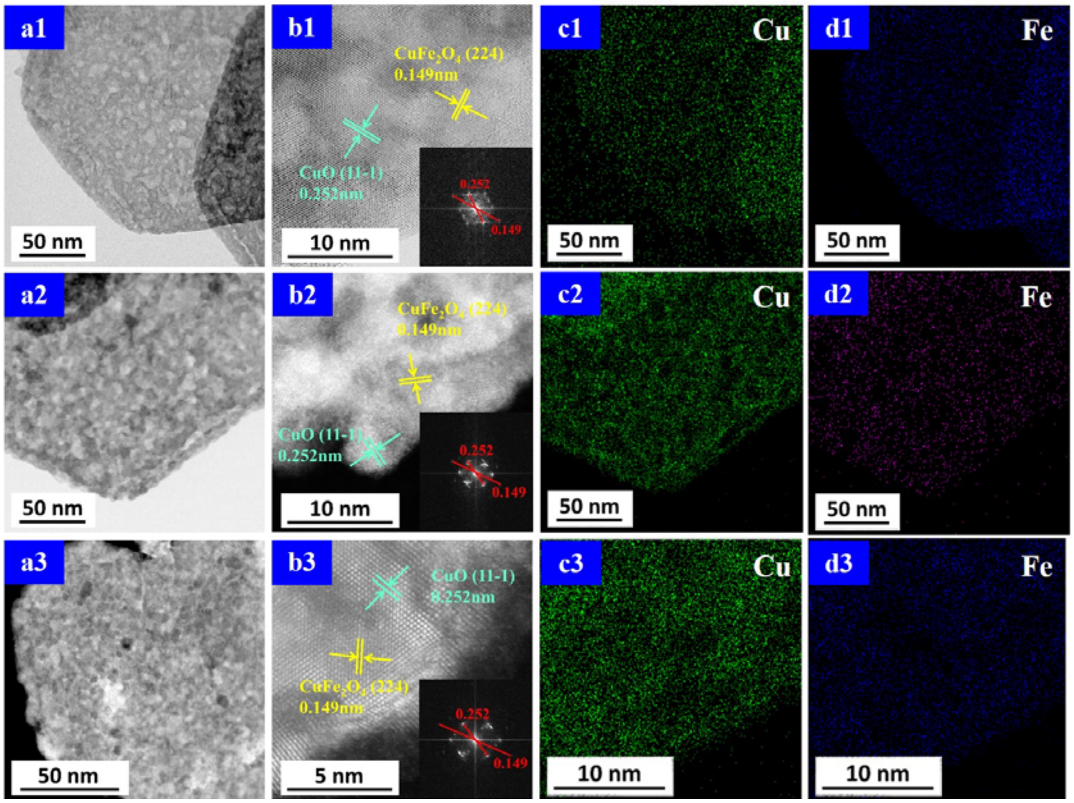

Supplementary Figure S3. (a1–a3) TEM images of $Cu_1Fe_1Mg_4$-MMO, $Cu_2Fe_1Mg_4$-MMO and

$Cu_4Fe_1Mg_4$-MMO. (b1–b3) HRTEM images selected from (a1–a3) and their Fourier transform patterns of the selected region (inset), respectively. (c1–c3) and (d1–d3) EDS mapping of elemental distribution for Cu and Fe.

• **p.4, Line 83:** rephrase: "According to the H_2-TPR measurements (Figure 1c), the main peak shifts gradually from 280 °C to 350 °C with the increase of Cu/Fe ratio, which is ascribed to the enhanced amount of $CuFe_2O_4$ spinel (a high reduction temperature) within CuO matrix (a low reduction temperature)[35-37], as confirmed by XRD (Figure 1b) and HRTEM (Figure S3)."

(3) For the catalyst activation, why 50% CO_2 is necessary to get better catalytic performance? According to Supplementary Table 4, catalyst reduction under 50% syngas+ 50% CO_2 brought in good enhancement of CO conversion and alcohols production. What role does it play during the activation procedure?

Author reply: We thank the reviewer for the comment. We found that calcination temperature (Table S2), activation procedure (Table S3) and activation atmosphere (Table S4) have influences on the production of long-chain alcohols synthesis. As for the activation process, previous studies have shown that the formation of iron carbide is mainly affected by temperature and gas phase composition (*J. Am. Chem. Soc.* **2010**, *132*, 14928; *J. Catal.* **2014**, *317*, 135). In this work, we have systematically tried syngas and syngas containing CO_2, and the results showed CO_2 mixed in syngas could slow down the rate of reduction and facilitate carburization of Fe to produce Fe_5C_2 species, which would maintain the tiny size of Fe_5C_2 to form abundant Cu-Fe_5C_2 interfacial sites.

• **p.6, Line 103:** rephrase: "The sample activated in syngas containing CO_2 (Table S4) could slow down the rate of reduction and facilitate carburization of Fe to produce Fe_5C_2 species, which would maintain the tiny size of Fe_5C_2 to form abundant Cu-Fe_5C_2 interfacial sites."

(4) Supplementary Table 3 looks very confusing. I believe the last row should be "300 °C 2h + 350 °C 1h". What's the meaning of 'c' and which entry is it for? Please double check this.

Author reply: We thank the reviewer for the comment. We have redesigned Table 3 and added the annotation ***c*** into entry 3.

Supplementary Table S3. Catalytic performances of samples with various activation steps

Entry[a,b]	Conv. [mol%]	Selectivity[mol%]				Alcohols distribution[%][c]				
		CH_4	$C_{2+}H$	ROH	CO_2	MeOH	EtOH	PrOH	BuOH	$C_{5+}OH$
500 °C 20 h	4.8	5.6	41.8	45.2	7.4	85.4(38.6)	1.9(0.8)	1.5(0.7)	0.8(0.4)	10.4(4.7)
350 °C 2 h	33.1	1.7	64.2	7.2	26.9	8.4(0.6)	18.4(1.3)	7.2(0.5)	1.3(0.1)	64.7(4.7)
350°C 10h (H_2:CO:CO_2:N_2=1:1:2:8, 100 ml min^{-1})	12.8	2.3	63.9	13.2	20.6	20.8(2.7)	25.3(3.3)	5.4(0.7)	1.2(0.2)	47.3(6.3)
300 °C 2 h + 350 °C 1 h	48.7	2.8	48.0	15.6	33.6	6.0(0.9)	31.7(4.9)	5.8(0.9)	2.5(0.4)	54.0(8.5)

[a] Activation conditions : $Cu_4Fe_1Mg_4$-MMO, 1 g precursor, H_2:CO:CO_2=1:1:2 (40 ml min^{-1}), 2 ºC min^{-1}.

[b] Reaction conditions: 3 MPa, 260 ºC, H_2/CO=2, 2400 mL g_{cat}^{-1} h^{-1}.

[c] Normalized data to S_{ROH}, mol% in brackets.

(5) For sample Cu1Fe1 and Cu2Fe1, CO conversion is increasing with pressure ascending, as listed in Supplementary Table 7, which is a normal phenomenon. However, CO conversion on Cu4Fe1 reduced with increased pressure. Why is a so big difference between Cu4Fe1 and the other two counterparts?

Author reply: We thank the reviewer for the comment. In this work, long-chain alcohols synthesis at 1 MPa was achieved, which was ascribed to the unique structure of Cu_4Fe_1 catalyst. The structural property imposes Cu_4Fe_1 catalyst with an appropriate activation ability toward CO and H_2, which promotes the matching degree of reaction rate between CO dissociation and C−C bond propagation. Corresponding discussions have been improved in the revised manuscript.

• **p.13, Line 204:** rephrase: "With Fe_1 as reference, these two peaks (440 °C and 490 °C) are attributed to interfacial Fe_5C_2 (Fe_5C_{2int}) and isolated Fe_5C_2 (Fe_5C_{2iso}) species, respectively. Clearly, Cu_6Fe_1 catalyst has the highest density of interfacial Fe_5C_2 sites. We fitted and deconvoluted the two peaks to roughly estimate the ratio of Fe_5C_{2int}/(Fe_5C_{2int}+ Fe_5C_{2iso}), and the results showed a volcanic correlation between the yield of long-chain alcohols and the relative concentration of interfacial Fe_5C_2. Cu_4Fe_1 catalyst possesses the highest concentration of Fe_5C_2-Cu interface sites (Fe_5C_{2int}), accounting for the largest long-chain alcohols yield (0.101 g g_{cat}^{-1} h^{-1}). This demonstrates that the Fe_5C_2−Cu interfacial sites act as active center toward LAS. "

• **p.14, Line 222:** rephrase: "Compared with other Cu_xFe_y samples, Cu_4Fe_1 shows the weakest ability toward H_2 adsorption and activation (Figure S13a). As the reaction pressure decreases from 3 MPa to 1 MPa, hydrogen activation on catalyst surface is weakened (Figure S13b), which reduces the rate of hydrogenation and hydrocarbons chain termination and thus enhances the growth of carbon-chain. In contrast, the CO activation is promoted since CO molecule is prone to adsorb on a Cu-rich surface[56,57]. This facilitates the kinetic rate matching between CO insertion and C−C coupling, and therefore significantly elevates the selectivity toward total alcohols and long-chain alcohols[58-60]. This is also verified by the α-ASF chain-lengthening probabilities analysis: the α-value for alcohols (0.72) at 1 MPa exceeds that for hydrocarbons (0.70), indicating the rate of CO insertion is larger than that of hydrocarbons chain termination (Figure S4). Therefore, a precise control over double-active-site in Cu_4Fe_1 catalyst accounts for the high yield toward long-chain alcohols at 1 MPa."

(6) In Page 13, line 216, the author mentioned Supplementary Figure 12, but this figure is not included in the supporting information.

Author reply: We thank the reviewer for the comment. We have corrected this error.

(7) Authors made the statement that excellent performance at a very low pressure of 1MPa, which is comparable to these conventional catalysts operated at least 3 MPa. Please make it clear that the comparison is based on similar operation temperature as these conventional catalysts.

Author reply: We thank the reviewer for the comment. We have supplemented catalytic performance data based on similar operation temperature (260 °C) as previous reports (Supplementary Table S9). The long-chain alcohols yield in our work is among the highest level, and takes the merit of low-pressure reaction (1 MPa) compared with previous studies (above 3 MPa).

Supplementary Table S9. Catalytic performance data for a variety of modified F−T catalysts used in LAS

Catalyst	H_2/CO ratio	Temperature /℃	Pressure /MPa	GHSV	CO conversion/%	Total alcohol Selectivity/%	Long-chain alcohol selectivity/%	Total alcohols STY[a]	Long-chain alcohols STY[a]	reference
**Cu_4Fe_1**	2	260	1	2400 mL g_{cat}^{-1} h^{-1}	53.2	29.8	49.1	0.201	0.101	This work
**CuZnFeMn**	2	260	4	6000 h^{-1}	52.62	31.04	3.65	0.24	0.016	*Catal. Commun.* **2008**, *9*, 1869-1873
**3DOM Cu_2Fe_1**	1	260	4.8	2000 h^{-1}	57.5	33.6	52.4	0.20	0.100	*ChemCatChem* **2014**, *6*, 473-478
**$CF_{0.5}$**	2	260	4	5000 h^{-1}	17.99	20.77	2.5	0.05	0.001	*J. Colloid Interface Sci.* **2016**, *470*, 162-171
**CNF-2-0.005**	1.5	240	5	32000 mL g_{cat}^{-1} h^{-1}	11	39		0.53		*ACS Catal.* **2018**, *8*, 9604-9618
**CuFe NPs**	2	220	6	6000 h^{-1}	17.1	21.9	64	0.14	0.085	*J. Mol. Catal. A: Chem.* **2013**, *378*, 319-325
**0.5%K-FeCuMnZnO**	2	260	4	6000 h^{-1}	27.3	49.3	3	0.29		*Appl. Energy* **2015**, *138*, 584-589
**CoGa-ZnAl-LDO/ Al_2O_3**	2	260	3	2000 h^{-1}	43.5	59	37.7	0.24	0.091	*J. Catal.* **2016**, *340*, 236-247
**CoMn | CuZnAlZr**	2	230	6	2000 mL g_{cat}^{-1} h^{-1}	17.8	46.1	57.1	0.04	0.023	*Angew. Chem., Int. Ed.* **2019**, *58, 4627-4631*
**CoCu/MoO_x**	1	270	4	120000 mL g_{cat}^{-1} h^{-1}	<2	46		0.03		*Angew. Chem., Int. Ed.* **2014**, *53*, 6397-6401
**$(Cu_1Co_2)_2$Al/CNT**	2	230	3	3900 mL g_{cat}^{-1} h^{-1}	45	62	13.8	0.34	0.05	*J. Mater. Sci.* **2016**, *51*, 5216-5231
**CuFeCo**	2	350	5.5	6000 h^{-1}	72	12.5	6	0.25	0.015	*Appl. Catal. A* **2015**, *503*, 51-61

**Fe/K/ Mo_2C(Fe/Mo= 1/14)**	2	320	7	4000 h^{-1}	50.25	22.69	0.62	0.14	0.001	*Catal. Lett.* **2010**, *136*, 9-13
**S2-CuFeMg-Cat**	2	300	4	2000 h^{-1}	56.89	49.07	11.25	0.28	0.032	*Catal. Sci. Technol.* **2013**, *3*, 1324-1332
**CuFeK0.5M**	2	320	5	6000 h^{-1}	53	61		0.32		*Fuel Process. Technol.* **2017**, *159*, 436-441
**Fe-CuMnZrO_2(I)**	2	310	8	8000 h^{-1}	45.5	26.2	3	0.45	0.014	*J. Mol. Catal. A: Chem.* **2004**, *221*, 51-58
**Fe-Cu/Al_2O_3 (Al_2O_3 loading: 89.3%)**	2.68	380	4	10000 h^{-1}		62.3		0.044		*J. Nat. Gas Chem.* **2008**, *17*, 327-331
**Cu-Fe-K-M_{80}**	2	320	5	6000 h^{-1}	56	63		0.35		*Energy Procedia* **2015**, *75*, 767-772
**$Cu_{20}Fe_{30}K_1M$**	2	320	5	6000 h^{-1}	46	53		0.35		*Catal. Today* **2014**, *234*, 278-284
**K–CoMo/(Co/CNT-h)**	2	320	5	10000 mL g_{cat}^{-1} h^{-1}	57	47	47	0.30	0.141	*Appl. Catal., A* **2008**, *340*, 87-97
**$Co_4Mn_1K_{0.1}$**	5	220	4	3600 h^{-1}	34	44	22	0.20	0.044	*Nat Commun.* **2016**, *7*, 13058-13064
**CoCuMn**	2	200	6	3600 h^{-1}	3	52	65	0.12	0.078	*J. Am. Chem. Soc.* **2013**, *135*, 7114-7117
**$Co_1Mo_1K_{0.05\text{-}12\%}$ (4.2% Co/MWCNT)**	2	290	5	8000 mL g_{cat}^{-1} h^{-1}	21.1	85		0.33		*Appl. Catal. A* **2008**, *340*, 87-97

[a] **g g_{cat}^{-1} h^{-1}**

Reviewer #3 (Remarks to the Author):

Wei and co-workers have studied CuFe catalysts for syngas conversion to higher alcohols. By starting from an LDH precursor they assure intimate contact between the copper and iron phases. They propose that carbon chains grow on the iron carbide phase while at the copper-iron interphase chain termination via CO insertion takes place. This is a very interesting idea together with reasonable activities and selectivities of the catalysts which in principle could warrant publication in Nature Communications. However, in my opinion several issues have to be worked out before a final decision can be taken.

(1) The paper contains many ambiguities. In fact one often has to guess about the meaning of symbols, units and procedures during measurements. Their description of Methods is too concise and I suggest that the make extensive descriptions of their procedures in the Supplementary Information. There are too many examples to cover here so I only mention three below (point 2-4).

Author reply: We thank the reviewer for the comment. We have provided detailed description on preparation methods in the revised manuscript.

• **p.16, Line 250:** rephrase: "General activation process: the $Cu_xFe_yMg_4$-LDH precursors were calcined in air at 500 °C for 4 h at a heating rate of 2 °C min^{-1} to obtain mixed metal oxides (MMOs) (denoted as $Cu_1Fe_1Mg_4$-MMO, $Cu_2Fe_1Mg_4$-MMO, $Cu_4Fe_1Mg_4$-MMO and $Cu_6Fe_1Mg_4$-MMO); subsequently, these MMOs materials were reduced in a gas atmosphere consisting of 25% CO + 25% H_2 + 50% CO_2 with a two-step-process—300 °C for 2 h and 350 °C for another 1 h at a heating rate of 2 °C min^{-1}, to obtain final catalyst samples (denoted as Cu_1Fe_1, Cu_2Fe_1, Cu_4Fe_1 and Cu_6Fe_1, respectively)."

• **p.17, Line 289:** rephrase: "The ASF chain growth probability α is calculated according to the equation: $\ln (W_n/n) = n\ln\alpha + \ln(1-\alpha)^2/\alpha$, where n is the number of carbon atoms in products; W_n is the weight fraction of products containing n carbon atoms; and $1-\alpha$ is the probability of chain termination."

• **p.19, Line 328:** rephrase: "The apparent kinetic order of H_2 was measured as follows: the catalytic test was performed at 260 °C with Cu_4Fe_1 catalyst (0.1 g) in a gas flow rate of 40 mL min^{-1} in order to keep the H_2 conversion under 10%. For determining the order of H_2, the partial pressure of H_2 was controlled from 22.5% to 45%."

(2) Selectivities are expressed in % or mol% without an explanation whether it is moles of products or (which is more common) in %C.

Author reply: We thank the reviewer for the comment. We have corrected the formula (2).

Product selectivity was defined as: Selectivity (mol %) = $\frac{F_{Ci} \times i}{\Sigma F_{Ci} \times i} \times 100$ (2)

(3) TEM and many other characterizations are applied without specification of the state of the catalyst. Was it calcined, reduced or reduced plus passivated?

Author reply: We thank the reviewer for the comment. We have added the catalyst state in illustration of all figures. Calcined sample means $Cu_xFe_yMg_4$-MMO obtained *via* calcination of $Cu_xFe_yMg_4$-LDH precursors. Reduced sample indicates Cu_xFe_y catalysts obtained through reducing $Cu_xFe_yMg_4$-MMO samples in 25% CO + 25% H_2 + 50% CO_2 atmosphere. Reduced plus passivated sample means a further passivation treatment of Cu_xFe_y catalysts in 5% O_2 + 95% N_2. This has been described in the revised manuscript and SI.

(4) What is semi-in-situ STEM? Not described what they have done.

Author reply: We thank the reviewer for the comment. The sample in this experiment was hold in glovebox in Ar atmosphere and transferred by a vacuum transfer TEM holder. By consulting related descriptions in previous reports, we have changed '*semi-in-situ*' into '*quasi-in-situ*'.

- **p.18, Line 299:** rephrase: "The *quasi-in-situ* scanning transmission electron microscopy (STEM) and EDX mapping measurements were performed on a FEI Tecnai G2 F20 microscope with an accelerating voltage of 120 kV. The sample was hold in glovebox in Ar atmosphere and transferred by a vacuum transfer TEM holder."

(5) In the Abstract the authors should report selectivities to higher alcohols in a precise manner.

Author reply: We thank the reviewer for the comment. We have addressed this issue in the Abstract.

- **p.2, Line 26:** rephrase: "The interfacial catalyst exhibits a CO conversion of 53.2%, a selectivity of 14.8 mol% and a space time yield of 0.101 g g_{cat}^{-1} h^{-1} for long-chain alcohols, with a surprisingly benign reaction pressure of 1 MPa."

(6) XRD is not based on 'reflection' but on 'diffraction'. So check the paper to change reflections into diffractions.

Author reply: We thank the reviewer for the comment. We have changed 'reflection' to 'diffraction' in the whole manuscript.

(7) Scientifically my main concern about this paper is that the authors provide little evidence that the chain termination can only take place on the interface between FeCx and Cu. I suggest that they vary the distance between pure Cu and Fe phases, e.g. by using physical mixtures of different intimacies of Cu and Fe. We know that in bifunctional catalysis the intimacy is key but that surface diffusion can take place and thereby direct contact between active phases is often not necessary.

Author reply: We thank the reviewer for the comment. We have supplemented three samples with different states between Cu and Fe phases. Cu_4/Fe_1 sample means a physical mixture of Cu_4 and Fe_1. Cu_4+Fe_1 sample denotes that pure Cu_4 and Fe_1 are located at the top and bottom of catalyst bed separated by an inert layer of quartz wool; and Fe_1+Cu_4 sample has a similar definition. When Cu and Fe phases are separated, long-chain alcohols can not be synthesized due to the absence of synergistic effect; and the main products are hydrocarbons and methanol.

• **p.8, Line 137:** rephrase: "The Cu_4 sample mainly shows methanol synthesis performance and the Fe_1 gives conventional FTS performance (Table S11), in accordance with previous work[38-40]. When separate Cu_4 and Fe_1 catalysts were combined with various modes, long-chain alcohols could not be synthesized. This indicates that the Cu-Fe_5C_2 synergistic effect is responsible for the production of long-chain alcohols."

Supplementary Table S11. Catalytic performances of samples with different preparation methods and composition

Catalysts[a,b]	Conv. [%]	Selectivity[mol%]				Alcohols distribution[%][c]				
		CH_4	$C_{2+}H$	ROH	CO_2	MeOH	EtOH	PrOH	BuOH	$C_{5+}OH$
Cu_4Fe_1	53.2	3.7	36.5	29.8	30.0	8.7(2.6)	28.8(8.5)	8.2(2.4)	5.2(1.5)	49.1(14.8)
Cu_4Fe_1-co	37.1	13.4	31.3	35.2	20.1	9.6(3.4)	79.6(28.0)	6.7(2.3)	2.4(0.8)	1.7(0.7)
Cu_4Fe_1-im	9.7	19.9	52.0	10.5	17.6	81.2(8.5)	9.6(1.0)	5.3(0.6)	3.9(0.4)	–
Cu_4	5.9	23.1	2.1	70.5	4.3	92.0(64.8)	3.9(2.7)	2.7(1.9)	1.4(1.1)	–
Fe_1	19.2	21.3	38.6	–	40.1	–	–	–	–	–
Cu_4/Fe_1[e]	7.8	25.5	21.7	27.6	25.2	85.6(23.6)	8.8(2.4)	5.6(1.6)	–	–
Cu_4+Fe_1[f]	6.2	21.7	47.8	15.0	15.5	89.1(13.3)	7.3(1.1)	3.6(0.6)		
Fe_1+Cu_4[g]	6.5	27.3	34.3	19.6	18.8	90.6(17.7)	7.6(1.5)	1.8(0.4)		

[a] Activation conditions: 1 g precursor, H_2: CO: CO_2=1: 1: 2 (40 mL min^{-1}), 2 ºC min^{-1}, 300 ºC 2 h + 350 ºC 1 h.
[b] Reaction conditions: 1 MPa, 260 ºC, H_2/CO=2, 2400 mL g_{cat}^{-1} h^{-1}.
[c] Normalized data to S_{ROH}, mol% in brackets.
[e] Physical mixture of Cu_4 and Fe_1
[f] Fe_1 is located separately below Cu_4 by an inert layer of quartz wool.
[g] Cu_4 is located separately below Fe_1 by an inert layer of quartz wool.

(8) The TEM work is not too convincing to me. For example in Figure 8 (SI) they focus on a large Cu particle to show that FeC is dispersed on or connected with the large Cu particle. What about the many bright dots in Figure 8-a that suggest many small Cu particles? Are these also in direct contact with FeC?

Author reply: We thank the reviewer for the comment. We have supplemented additional TEM images with various magnification and selected area in Supplementary Figure S8 and S9, to give a clear illustration.

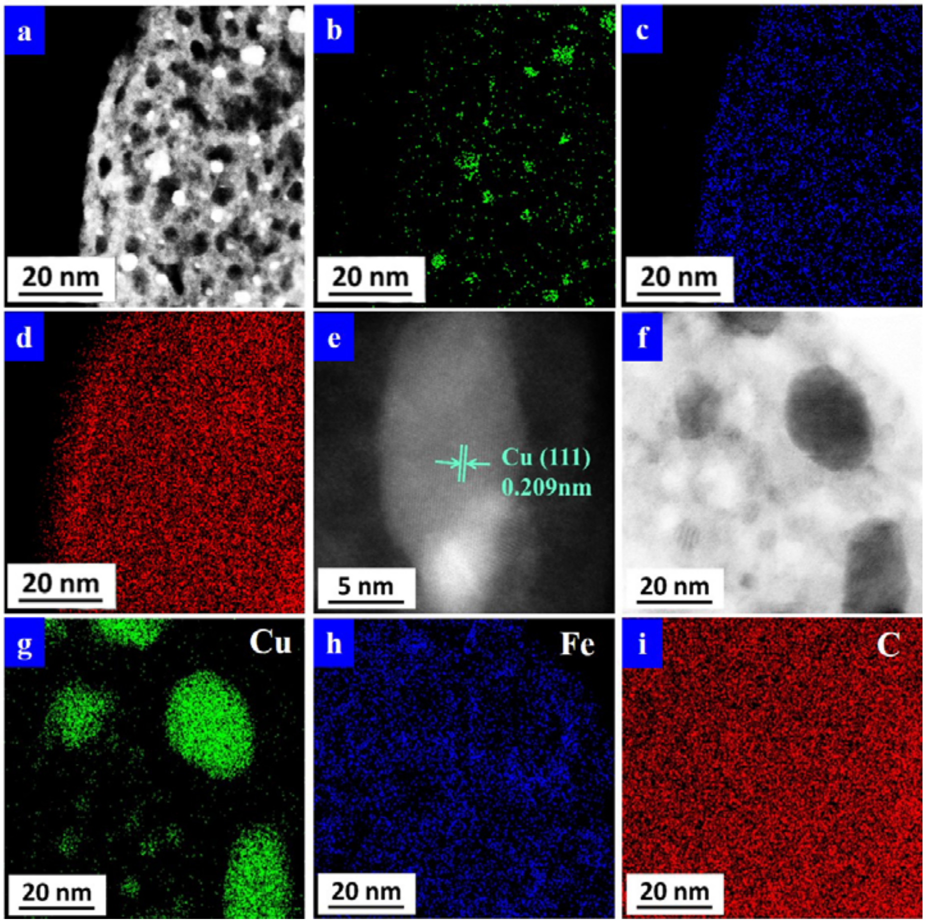

Supplementary Figure S8. (a, e and f) TEM images of Cu_1Fe_1 catalyst (reduced and passivated). (b, c, d, g, h and i) EDS mapping of elemental distribution for Cu, Fe and C, respectively.

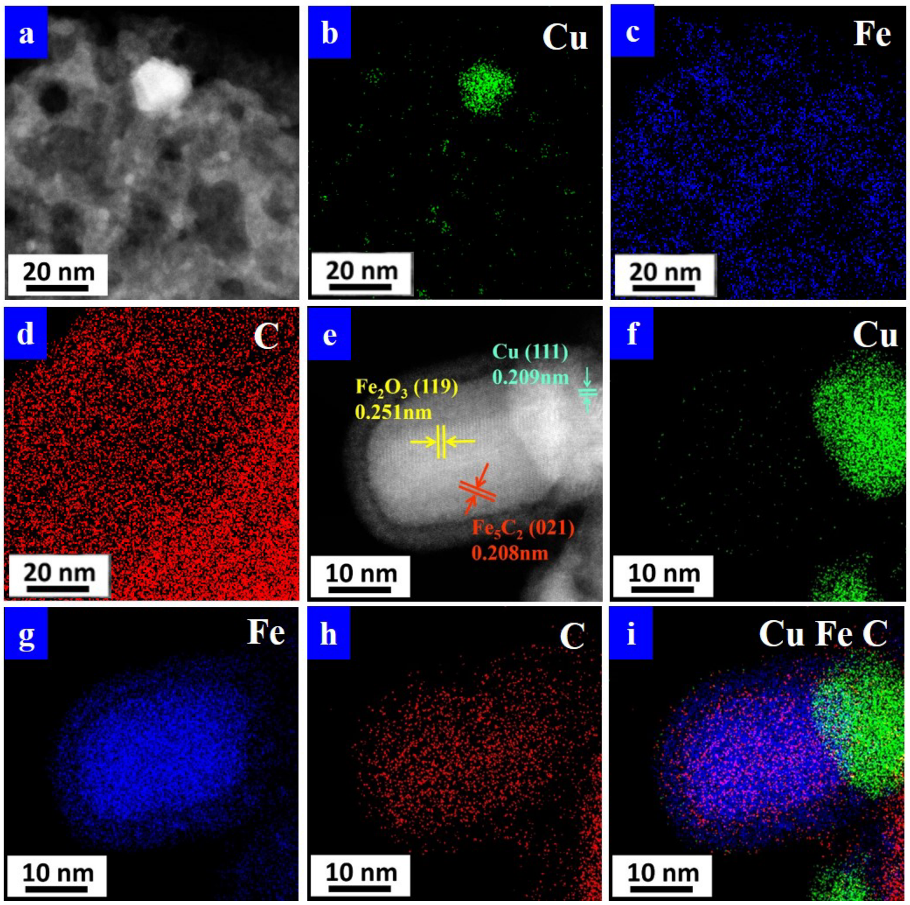

Supplementary Figure S9. (a, e) TEM images of Cu_2Fe_1 catalyst (reduced and passivated). (b, c, d, f, g, h and i) EDS mapping of elemental distribution for Cu, Fe and C, respectively.

• **p.9, Line 151:** rephrase: "For Cu_1Fe_1 and Cu_2Fe_1 samples (Figure S8 and S9), the lattice fringe of Cu (111) with 0.209 nm is clearly resolved; EDS mapping images of Cu, Fe and C demonstrate the existence of Fe_5C_2 nanoclusters on the surface of Cu nanoparticles."

In conclusion, very interesting study but more rigor is needed in writing and in establishing the Fe-Cu interface as the cause for higher alcohol formation.

REVIEWERS' COMMENTS:

Reviewer #1 (Remarks to the Author):

The revised manuscript has considerably improved, but there are a few relevant points that should be tackled before it could be published.

The discussion of the Cu-Fe interplay determining chain length is still not convincing. I do agree that a low interface should not allow production of alcohols higher than methanol, as demonstrated with the new physical mixture and series bed. Still, high interface should maximize the selectivity towards short alcohols since CO insertion will terminate chains easily and medium interface should improve that towards long alcohols since more time is given to the chains to grow before CO insertion. This holds assuming that the no other property in the catalysts is changed besides for the interfacial density. To explain that high interface leads to long-chain alcohols the role of other parameters should be better elucidated.

'Clearly, Cu6Fe1 catalyst has the highest density of interfacial Fe5C2 sites. … Cu4Fe1 catalyst possesses the highest concentration of 214 Fe5C2-Cu interface sites (Fe5C2int), accounting for the largest long-chain alcohols yield (0.101 g 215 gcat−1 h−1). This demonstrates that the Fe5C2−Cu interfacial sites act as active center toward LAS.' Please amend this contradictory text. Additionally interfacial sites are good for higher alcohols synthesis in general. The Cu4Fe1 catalyst is intrinsically more active. This is why the STY to LAS over this material is the highest. This is obvious when looking at Figures 2a and 2b. The Cu6Fe catalyst is more selective to LAS than Cu4Fe but less active. This point should be elucidated using experiments to determine selectivity at iso-conversion, which I have already asked for in the first revision. Results for Cu1-4Fe have been added to the SI but have not been discussed. Clearly data for Cu6Fe would have been relevant too. They should be used to compare the catalyst instead of the data currently used to build Figure 2a. The paper should discuss the interplay of activity and selectivity that leads to the maximal STY of LAS. The activity changes because the relative particle sizes of Cu and iron carbides have not been kept constant, as one should have done in a more elegant study.

'This facilitates the kinetic rate matching between CO insertion and C−C coupling, and therefore significantly elevates the selectivity toward total alcohols and long-chain alcohols.' This concept is wrong. If you match the rates of CO insertion and of C-C coupling you would only produce ethanol and ethane, not long-chain alcohols. Chain growth should be favored over insertion to allow the chain to elongate before is terminated.

What does ‚selectivity of 14.8 mol%' mean? Does it correspond to 14.8%? Besides, what is the error in the measurements? Are decimal digits significant, or should the values be rounded to integer numbers?

Long-chain alcohols has been first abbreviated with LAS. Then LA is used. Please be consistent.

Reviewer #2 (Remarks to the Author):

Authors have made a great effort to address the issues raised by the referees and improve the quality of the paper accordingly. I advise that the paper can be accepted.

Reviewer #3 (Remarks to the Author):

Manuscript has been properly revised and can be accepted as is.

Response to Reviewers

Reviewer #1:

Reviewer #1 (Remarks to the Author):

The revised manuscript has considerably improved, but there are a few relevant points that should be tackled before it could be published.

(1) The discussion of the Cu-Fe interplay determining chain length is still not convincing. I do agree that a low interface should not allow production of alcohols higher than methanol, as demonstrated with the new physical mixture and series bed. Still, high interface should maximize the selectivity towards short alcohols since CO insertion will terminate chains easily and medium interface should improve that towards long alcohols since more time is given to the chains to grow before CO insertion. This holds assuming that the no other property in the catalysts is changed besides for the interfacial density. To explain that high interface leads to long-chain alcohols the role of other parameters should be better elucidated.

Author reply: We thank the reviewer for this comment. According to previous studies (*Nat. Commun.* **2016**, *7*, 13058; *Angew. Chem. Int. Ed.* **2014**, *53*, 6397; *Angew. Chem. Int. Ed.* **2019**, *58*, *4627*) as well as our experimental results, we consider that the high yield of long-chain alcohols over Cu_4Fe_1 catalyst can be attributed to the interplay of activity and selectivity. The Fe_5C_2-Cu interface with a high ratio would increase long-chain alcohols selectivity, which is shown in Figure 2a and Supplementary Table S7. In addition, the unique interface structure of Cu_4Fe_1 would promote the kinetic rate coordination between CO insertion and C–C coupling to maintain a high selectivity toward long-chain alcohols under low pressure. Corresponding discussions have been improved in the revised manuscript.

• **p.14, Line 224:** rephrase: "The Fe_5C_2 nanoclusters, with an ultra-small size (normally below 2 nm) on the surface of Cu nanoparticles, provide active sites for CO activation/dissociation and the resulting C–C bond propagation, which maintains the high activity of Cu_4Fe_1. As the reaction pressure decreases from 3 MPa to 1 MPa, hydrogen activation on catalyst surface is weakened (Supplementary Figure 13), which reduces the rate of hydrogenation and hydrocarbons chain termination and thus enhances the growth of carbon-chain. In contrast, the CO activation is promoted since CO molecule is prone to adsorb on a Cu-rich surface[56,57]. This facilitates the kinetic rate coordination between CO insertion and C–C coupling, and therefore significantly elevates the selectivity toward total

alcohols and long-chain alcohols[58-60]. This is also verified by the α-ASF chain-lengthening probabilities analysis: the α-value for alcohols (0.72) at 1 MPa exceeds that for hydrocarbons (0.70), indicating the rate of CO insertion is larger than that of hydrocarbons chain termination (Supplementary Figure 4). Therefore, a precise control over double-active-site in Cu_4Fe_1 catalyst accounts for the high yield toward long-chain alcohols at 1 MPa."

(2) "Clearly, Cu_6Fe_1 catalyst has the highest density of interfacial Fe_5C_2 sites. … Cu_4Fe_1 catalyst possesses the highest concentration of Fe_5C_2-Cu interface sites (Fe_5C_{2int}), accounting for the largest long-chain alcohols yield (0.101 g g_{cat}^{-1} h^{-1}). This demonstrates that the Fe_5C_2−Cu interfacial sites act as active center toward LAS." Please amend this contradictory text. Additionally interfacial sites are good for higher alcohols synthesis in general. The Cu_4Fe_1 catalyst is intrinsically more active. This is why the STY to LAS over this material is the highest. This is obvious when looking at Figures 2a and 2b. The Cu_6Fe catalyst is more selective to LAS than Cu_4Fe but less active. This point should be elucidated using experiments to determine selectivity at iso-conversion, which I have already asked for in the first revision. Results for $Cu_{1-4}Fe$ have been added to the SI but have not been discussed. Clearly data for Cu_6Fe would have been relevant too. They should be used to compare the catalyst instead of the data currently used to build Figure 2a. The paper should discuss the interplay of activity and selectivity that leads to the maximal STY of LAS. The activity changes because the relative particle sizes of Cu and iron carbides have not been kept constant, as one should have done in a more elegant study.

Author reply: We thank the reviewer for the comment. According to this comment, we supplemented catalytic evaluation over Cu_4Fe_1 and Cu_6Fe_1 iso-conversion, in comparison with Cu_1Fe_1 and Cu_2Fe_1 through altering WHSV (see Figure 2a and Supplementary Table S7). The alcohols distribution over Cu_4Fe_1 and Cu_6Fe_1 with iso-conversion (~30%) by altering WHSV is added in Figure 2a. Corresponding discussions have been improved in the revised manuscript.

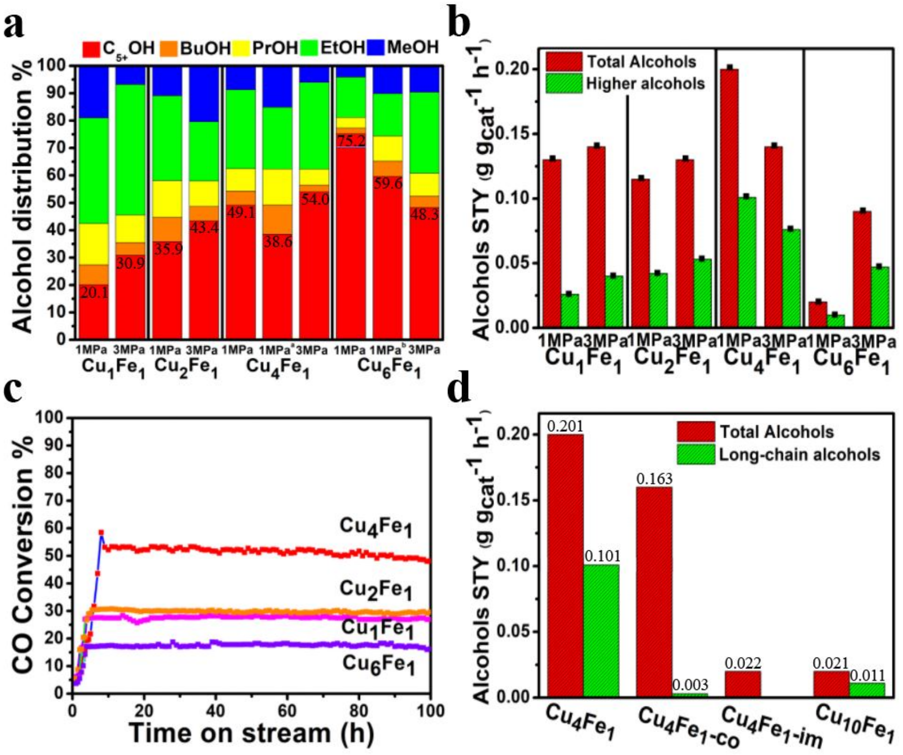

Fig. 2 Catalytic performance. (a) Alcohol distribution normalized data to total alcohols selectivity ([a] WHSV = 4800 mL g_{cat}^{-1} h^{-1}; [b] WHSV = 1200 mL g_{cat}^{-1} h^{-1}; $C_{5+}OH$: long-chain alcohols, BuOH: butanol, PrOH: propanol, EtOH: ethanol and MeOH: methanol) and (b) alcohols STY at different pressures over catalysts with four Cu/Fe ratios (1/1, 2/1, 4/1 and 6/1) (reaction conditions: 27% CO + 55% H_2 + 18% N_2; 260 °C; WHSV of 2400 mL g_{cat}^{-1} h^{-1}). (c) Time-on-stream (TOS) evolution of CO conversion over the four catalysts within 100 h test at 1 MPa. (d) Alcohols STY of Cu_4Fe_1, Cu_4Fe_1-co, Cu_4Fe_1-im and $Cu_{10}Fe_1$ at 1 MPa.

• **p.7, Line 123:** rephrase: “The Cu_4Fe_1 has 38.6% at WHSV = 4800 mL g_{cat}^{-1} h^{-1} with iso-conversion to other Cu_xFe_y samples, although Cu_4Fe_1 has higher value. The total alcohols yield (0.201 g g_{cat}^{-1} h^{-1}) of Cu_4Fe_1 is much higher than hydrocarbons yield (0.111 g g_{cat}^{-1} h^{-1}), demonstrating the predominant production of alcohols rather than hydrocarbons (Supplementary Table 8). This means the high LA yield is the interplay of activity and selectivity.”

• **p.13, Line 210:** rephrase: “Clearly, Cu_4Fe_1 gives the largest integral peak area, indicating the most abundant total Fe_5C_2 sites than other samples. We fitted and deconvoluted the two peaks to roughly estimate the ratio of Fe_5C_{2int}/(Fe_5C_{2int}+ Fe_5C_{2iso}) for these four Cu_xFe_y

catalysts, and the results showed a volcanic correlation between the LA yield and the relative concentration of interfacial Fe_5C_2 (Figure 4h). Cu_4Fe_1 catalyst with a moderate Fe_5C_{2int} ratio possesses the highest concentration of Fe_5C_2-Cu interface sites (Fe_5C_{2int}), accounting for the largest LA yield (0.101 g g_{cat}^{-1} h^{-1}). This demonstrates that the Fe_5C_2–Cu interfacial sites act as active center toward LA production."

(3) "This facilitates the kinetic rate matching between CO insertion and C−C coupling, and therefore significantly elevates the selectivity toward total alcohols and long-chain alcohols." This concept is wrong. If you match the rates of CO insertion and of C−C coupling you would only produce ethanol and ethane, not long-chain alcohols. Chain growth should be favored over insertion to allow the chain to elongate before is terminated.

Author reply: We thank the reviewer for the comment. This sentence has been revised as suggested.

- **p.14, Line 232:** rephrase: "This facilitates the kinetic rate coordination between CO insertion and C–C coupling, and therefore significantly elevates the selectivity toward total alcohols and long-chain alcohols[58-60]."

(4) What does, selectivity of 14.8 mol% mean? Does it correspond to 14.8%? Besides, what is the error in the measurements? Are decimal digits significant, or should the values be rounded to integer numbers?

Author reply: We thank the reviewer for the comment. In this work, the selectivity of 14.8 mol% corresponds to 14.8%, which was calculated based on the formula (2).

Product selectivity was defined as: Selectivity (mol %) = $\frac{F_{Ci} \times i}{\Sigma F_{Ci} \times i} \times 100$ (2)

In order to acquire accurate results of conversion and selectivity, the catalytic performance data were precisely calculated to one decimal place rounded from two decimal places by internal standard method of GC. Meanwhile, it is also significant for obtaining high carbon balance and mass balance, which would give larger error if the data are rounded to integer numbers.

(5) Long-chain alcohols has been first abbreviated with LAS. Then LA is used. Please be consistent.

Author reply: We thank the reviewer for the comment. In this work, the LA is the abbreviation of long-chain alcohols; while the LAS denotes long-chain alcohols synthesis. We have checked the manuscript carefully to make a consistency.

Reviewer #2:

Authors have made a great effort to address the issues raised by the referees and improve the quality of the paper accordingly. I advise that the paper can be accepted.

Author reply: We thank the reviewer for the comment.

Reviewer #3 (Remarks to the Author):

Manuscript has been properly revised and can be accepted as is.

Author reply: We thank the reviewer for the comment.
